# Genomic, Molecular Evolution, and Expression Analysis of Genes Encoding Putative Classical AGPs, Lysine-Rich AGPs, and AG Peptides in *Brassica rapa*

**DOI:** 10.3389/fpls.2017.00397

**Published:** 2017-03-29

**Authors:** Tianyu Han, Heng Dong, Jie Cui, Ming Li, Sue Lin, Jiashu Cao, Li Huang

**Affiliations:** ^1^Laboratory of Cell and Molecular Biology, Institute of Vegetable Science, Zhejiang UniversityHangzhou, China; ^2^Key Laboratory of Horticultural Plant Growth, Development and Quality Improvement, Ministry of AgricultureHangzhou, China; ^3^Zhejiang Provincial Key Laboratory of Horticultural Plant Integrative BiologyHangzhou, China; ^4^Institute of Vegetable Science, Wenzhou Vocational College of Science and TechnologyWenzhou, China

**Keywords:** AGPs, arabinogalactan proteins, phytohormones, pollen, syntenic analysis

## Abstract

Arabinogalactan proteins (AGPs) belong to a class of Pro/Hyp-rich glycoproteins and are some of the most complex types of macromolecules found in plants. In the economically important plant species, *Brassica rapa*, only chimeric AGPs have been identified to date. This has significantly limited our understanding of the functional roles of AGPs in this plant. In this study, 64 *AGPs* were identified in the genome of *B. rapa*, including 33 classical AGPs, 28 AG peptides and three lys-rich AGPs. Syntenic gene analysis between *B. rapa* and *A. thaliana* suggested that the whole genome triplication event dominated the expansion of the *AGP* gene family in *B. rapa*. This resulted in a high retained proportion of the *AGP* family in the *B. rapa* genome, especially in the least fractionated subgenome. Phylogenetic and motif analysis classified the classical AGPs into six clades and three orphan genes, and the AG peptides into three clades and five orphan genes. Classical AGPs has a faster rate of molecular evolution than AG peptides revealed by estimation of molecular evolution rates. However, no significant differences were observed between classical AGPs and lys-rich AGPs. Under control conditions and in response to phytohormones treatment, a complete expression profiling experiment has identified five anther-specific *AGPs* and quite a number of *AGPs* responding to abscisic acid, methyl jasmonate and/or gibberellin. In this study, we presented a bioinformatics approach to identify important types of *AGPs*. Moreover, the association between their function and their protein structure, as well as the evolution and the expression of *AGP* genes were investigated, which might provide fundamental information for revealing the roles of AGPs in *B. rapa*.

## Introduction

Arabinogalactan proteins (AGPs), together with proline-rich proteins (PRPs) and extensins (EXTs), constitute the superfamily of proline/hydroxyproline-rich glycoproteins (P/HRGPs), which are abundant in the extracellular matrix throughout the plant kingdom (Gaspar et al., 2001; Showalter, 2001). AGPs function in diverse aspects of plant growth and development, such as microspore embryogenesis (Tang et al., 2006), cell wall strengthening (Hijazi et al., 2014), cell proliferation (Serpe and Nothnagel, 1994), cell expansion (Willats and Knox, 1996), programmed cell death (Pennell et al., 1991), pollen tube growth (Levitin et al., 2008; Lin et al., 2014; Pereira et al., 2016), cell separation in vestigial abscission (Stenvik et al., 2006), and signal recognition and transduction (Lee et al., 2008, 2009). In addition, small number of initial studies have illustrated that AGPs are involved in response to phytohormones such as abscisic acid (ABA), methyl jasmonate (MeJA), and gibberellin (GA). For example, *AGP30* specifically responds to the concentration of ABA and then affects seed germination in *Arabidopsis thaliana* (van Hengel and Roberts, 2003). *AGP31* is down-regulated in MeJA treated *A. thaliana* plants (Liu and Mehdy, 2007), and some AGPs affect the expressions of gibberellin-induced genes in barley (Mashiguchi et al., 2008).

The AGP family consists of different members that can be variable in specific plant species. Therefore, genome-wide screens are commonly used for the identification of AGPs. Knowledge of distinct characteristics shared across known members of the protein family enables their detection within the complete set of proteins in an organism. AGPs are rich in proline or hydroxyproline (Pro/Hyp), serine (Ser), threonine (Thr), and alanine (Ala), which comprise up to 99% of the molecular mass of AGP proteins (Ellis, 2010). According to differences in the composition of their protein backbone, AGPs are further classified into classical AGPs, arabinogalactan (AG) peptides, lysine (Lys)-rich AGPs, and chimeric AGPs (Schultz et al., 2002). Classical AGPs are defined by the core protein containing Hyp, Ala, Ser, Thr, and Glycine (Gly) as the major amino acid constituents, and their C terminus is glycosylphosphatidylinositol (GPI) anchored (Showalter et al., 2010). Lys-rich AGPs have a Lys-rich domain of approximately 16 amino acid residues that is flanked on both sides by AGP glycol modules. AG peptides are composed of only 10–13 amino acid residues and the putative cell adhesion molecules (Schultz et al., 2002). Most AGPs are characterized by the entire protein containing only P/HRGP modules, while chimeric AGPs are consisted of one or two known P/HRGP motifs and additional unrelated motifs such as, fascilin-like domain for fasciclin-like AGPs (FLAs), early nodulin-like domain for eNod-like AGPs (ENODL) and non-specific lipid transfer protein-like domain for nsLTP-like AGPs (Schultz et al., 2002). Based on specific characteristics of the protein backbone and length, the presence of Ala-Pro, Pro-Ala, Ser-Pro, or Thr-Pro repeats, signal peptide and GPI anchor addition sequence, 22 classical AGPs, 16 AG peptides and three lys-rich AGPs were identified in the *A. thaliana* genome (Showalter et al., 2010) and 11 classical AGPs, 15 AG peptides and two lys-rich AGPs were found in the rice (*Oryza sativa*) genome (Ma and Zhao, 2010). In vegetable crops, chimeric AGPs like FLAs and ENODLs have been identified in *Brassica rapa* (Jun and Xiaoming, 2012; Li et al., 2013), which is the most important economic vegetable crop in East Asia (Wang et al., 2012). *B. rapa* shares a common ancestor with *A. thaliana*; their chromosomes were derived from the rearrangement of 24 conserved collinear blocks in ancestral karyotype of crucifer species (Cheng et al., 2013). A whole genome triplication (WGT) event, occurred 13–17 million years ago, has differentiated the genome of *B. rapa* from that of *A. thaliana*. This makes *B. rapa* an excellent system for studying the expansion of gene families (Wang et al., 2011). Chromosomal localization and gene duplication analysis illustrated that the expansion of FLAs and ENODLs in *B. rapa* depends on the WGT event, and several *FLAs* in *B. rapa* display similar expression patterns as their orthologs in *A. thaliana*. In our previous studies, a screening for differentially expressed genes between the male fertile and sterile plants of the *B. rapa* genic male sterility (GMS) A/B line has identified two classical AGPs encoding genes exclusively expressed in the fertile flower buds (Huang et al., 2008a). Further functional characterization revealed that these two AGPs might play important roles in pollen wall formation (Huang et al., 2008b; Lin et al., 2014). However, it is still unknown whether there are more classical AGPs function in pollen development in *B. rapa*. Also, there is limited information about the presence of other types of AGPs in *B. rapa*, which restricts a comprehensive understanding of the roles of *AGP* gene family in this crop.

In this study, in order to obtain more detailed information about the AGP family in *B. rapa*, Perl scripts were specifically written to screen for classical AGPs, AG peptides and lys-rich AGPs in the genome of *B. rapa*. Subsequently, the candidate *B. rapa AGPs* (*BrAGPs*) were annotated and their characteristics, including retained proportion, syntenic relationship, protein motif structure and molecular evolutionary rates, were analyzed. To understand the functions of BrAGPs and identify the candidate AGPs involved in pollen development, the expression of *AGP* genes in different tissues and different developmental stages of pollen were investigated. Furthermore, the response of *BrAGPs* to GA, MeJA and ABA treatments were also evaluated. This study may provide valuable insights and reveal some underlying mechanisms of the *AGP* gene family in *B. rapa*.

## Materials and methods

### Plant materials

The “*Bcajh97-01A/B*” is a GMS A/B line (sister line) of *B. rapa*, with the progenies segregating into sterile and fertile types in a 1:1 ratio. The sterile “*Bcajh97-01A*” has been demonstrated as a mutant of male meiosis cytokinesis without mature pollen, which is the only difference detected between “*Bcajh97-01A*” and the fertile “*Bcajh97-01B*” (Huang et al., 2008a, 2009). This characteristic of “*Bcajh97-01A/B*” GMS line is ideal to study gene expression during pollen development.

“*Bcajh97-01A/B*” was planted in experimental green-house of Zhejiang University. At the flowering stage, siliques at 48 h after pollination (HAP), roots, stems, leaves, and inflorescences were collected from 15 individual plants of the fertile line for reverse transcription PCR. At the same time, according to the longitudinal diameters, the flower buds were divided into five stages corresponding to five distinct pollen developmental stages (Huang et al., 2008a). The pollen development in flower buds was confirmed by microscopic examination.

Pistils in the fertile plants were collected at 1, 3, and 10 HAP. One HAP pistils correspond to the period of pollen germination on the stigma, 3 HAP pistils represent pollen tube growth phase, and 10 HAP pistils correspond to the period when the pollen tube reaches the ovule and fertilization occurs (Jiang et al., 2013). The materials were frozen in liquid nitrogen immediately after harvested and stored at −75°C for subsequent analysis.

### Phytohormone treatments

For phytohormone treatments, one of the following solutions of 100 μM GA, 100 μM MeJA, and 100 μM ABA was sprayed on all leaves (Liu et al., 2014; Duan et al, 2015). Control plants were sprayed with distilled water. The concentration of phytohormone treatments chosen was based on the studies by Suzuki et al. (2002), Liu and Mehdy (2007), and van Hengel et al. (2004). Seedlings in three containers were used in each treatment, and each container had 12 seedlings. The treatments were repeated three times. Control and treated leaves were harvested at 0, 4, and 12 h after treatment (HAT). The samples were frozen in liquid nitrogen and stored at −80°C until use.

### RNA isolation and reverse transcription PCR

Total RNA was extracted from tissues mentioned above using Trizol reagent (Invitrogen, Carlsbad, CA). The RNA integrity was analyzed on agarose gel. SuperScript IV (Invitrogen) and oligo (dT) primer was used to synthesize the cDNA. The expression of cDNA was detected using BiometraT Professional Thermo cycler and Taq (Dingguo, Shanghai). Reverse transcription polymerase chain reaction (RT-PCR) was employed to detect the expression of *BrAGPs* in different tissues of *B. rapa*, while quantitative real-time PCR (qRT-PCR) was carried out to test the expression variation of *BrAGPs* in treatment with endogenous hormones. RT-PCR and qRT-PCR were both carried out in triplicates using gene specific primers (Supplementary data 1: Tables S1, S2). *BrUBC10* was used as the reference gene. The results of qRT-PCR were calculated using the 2^−ΔΔCt^ method (Livak and Schmittgen, 2001) and normalized to the corresponding distilled water treatment. They were further gene-wise normalized, mean-centered and clustered hierarchically using the centroid linkage clustering method in Cluster 3.0 (http://bonsai.hgc.jp/~mdehoon/software/cluster/index.html).

### Identification of AGPs, AG-peptides and lys-rich AGPs by calculating the biased amino acid composition and length

The searching criteria for *AGPs* referred to the guidelines that are widely used (Schultz et al., 2002, 2004; Tan et al., 2003; Ma and Zhao, 2010). A Perl script (amino acid bias) was written to calculate the PAST (Pro, Ala, Ser, Thr) percentage for the candidate proteins (Supplementary data 1: Supplement Methods). All annotated proteins were downloaded from the Brassica database (BRAD, http://brassicadb.org, V1.2). Perl is a high-level, general-purpose, interpreted, dynamic programming language for UNIX, Windows, and OSX operating systems (http://www.activeperl.com/). For this study, the Perl language compiler ActivePerl-5.16.2.3010812913 was used.

The amino acid bias program was compiled with two different output files of the lists of proteins. The first output file named “AGPs” includes that all the proteins above 50% PAST threshold and >90 amino acid residues in length. Another output file named “AG-peptide” includes all proteins above 35% PAST and between 50 and 90 amino acid residues in length. The N-terminal and C-terminal signals of AG-peptide account for more than 50% of the deduced proteins and the length of AG-peptide is shorter than classical AGPs, so we reduced the PAST level and shortened the searching length of the sequences. Classical AGPs and lys-rich AGPs were extracted from file “AGPs,” in which lys-rich AGPs had a lys-rich domain of approximately 16 amino acid residues that was flanked on both sides by AGP glycomodules, and classical AGPs covered the other genes without the domains like fascilin-like domain and early nodulin-like domain. Pfam (http://pfam.xfam.org/) and SMART (http://smart.embl-heidelberg.de/) were used for analyzing the conserved domains. Moreover, orthologous genes of all the annotated classical AGPs, AG peptides and lys-rich AGPs in *A. thaliana* were screened in the proteome of *B. rapa*.

### Signal peptide and GPI anchor prediction

The signal peptide of proteins was predicted by the SignalP 4.1 Server (http://www.cbs.dtu.dk/services/SignalP/) (Petersen et al., 2011). In addition, proteins with predicted signal peptide cleavage site in the first 50 amino acids were considered as the presence of N-terminal signal sequences (Nielsen et al., 1997). To determine the presence of a C-terminal GPI anchor additional signal, all the proteins in the “AGPs” and “AG-peptide” files were subjected to the big-PI Plant Predictor (http://mendel.imp.ac.at/gpi/plant_server.html) and PSORT Prediction (http://psort.hgc.jp/form.html) (Nakai and Horton, 1999; Eisenhaber and Eisenhaber, 2007). Proteins having both signal peptide and GPI anchor were treated as “high confidence” AGPs.

### The analysis of synteny and retained proportion

The analysis of synteny using information of 24 conserved collinear blocks of ancestral karyotype (AK) in *B. rapa* and *A. thaliana* was carried out according to the previous study (Cheng et al., 2012, 2013). Chromosomal locations of the syntenic genes of *A. thaliana AGPs* (*AtAGPs*) and *BrAGPs* were gathered from The Arabidopsis Information Resource (TAIR, http://arabidopsis.org) and BRAD, respectively. The syntenic relationships between or inside the genomes were illustrated by Circos 5.05 (Krzywinski et al., 2009).

A set of 458 core eukaryotic genes and 458 random genes in *A. thaliana* (required for retained proportion analysis), were downloaded from CEGMA (http://korflab.ucdavis.edu/Datasets/cegma) (Parra et al., 2007) and were used to search for syntenic genes in the BRAD database. The orthologous genes of several *AtAGPs* in *A. lyrata, Capsella rubella, Camelina sativa, B. napus, B. oleracea, Thellungiella halophile, Thellungiella salsuginea, Sisymbrium irio, Schrenkiella parvula*, and *Aethionema arabicum* were detected through syntenic analysis in BRAD.

### Phylogenetic and protein motifs analysis

Multiple sequence alignment was performed by ClustalW (http://www.ch.embnet.org/software/ClustalW.html) in standard settings with some modification. Phylogenetic trees were constructed with both neighbor-joining (NJ) and maximum likelihood (ML) method with 1,000 bootstraps using MEGA6 software (Tamura et al., 2013). Poisson model were used in Model/Method and Uniform rates were chosen in Rates among sites. Treatment of Gaps/Missing Data was selected as Pairwise deletion. The clans consisting only one orthologous gene were treated as orphan genes, while other clans were named as clades in sequence. Motif analysis was performed in Multiple Em for Motif Elicitation (MEME) (http://meme-suite.org/tools/meme) (Bailey et al., 2009) for the identification of the short conserved motifs in the subfamilies of classical AGPs and AG peptides.

### Molecular evolutionary rate analysis

The molecular evolutionary rates between orthologous genes were estimated by calculating the ratio of nonsynonymous substitution rate (Ka) and synonymous substitution rate (Ks) between orthologous gene pairs. The protein sequences were aligned by ClustalW and the resulting alignment was used as a reference to align the nucleotide sequences. The Ka/Ks value was then determined using the YN86 module in PAML 4.7 (Yang, 2007). Significant differences were calculated by one-way ANOVA tests in SPSS 19 (IBM, USA).

## Results

### Identification of AGPs in the *Brassica rapa* genome

Initially, 76 proteins longer than 90 amino acids were retrieved in the *B. rapa* genome, with biased amino acid compositions of at least 50% PAST. Similarly, 115 potential AG peptides were identified by searching for proteins between 50 and 90 amino acids in length with biased amino acid compositions of at least 35% PAST (Table 1).

**Table 1:** **AGPs and AG peptides identified in the *Brassica rapa* genome based on amino acid composition, length and specific domains**.

**Search critieria**	**Total**	**SP^a^ & GPI^b^**	**Classical AGPs**	**AG peptides**	**Lys-rich AGPs**	**Others**
PAST^c^ >50% & length >90	76	58	22	0	3	33
PAST>35% & 50 < length <90	115	24	0	24	0	0

a SP, secretion peptide. N-terminal secretion signal peptides were predicted by SignalP;

b GPI was predicted both by big-PI Plant Predictor and PSORT Prediction;

c *PAST, the proportion of Pro, Ala, Ser, and Thr*.

Signal peptides are important for glycosylation of AGPs in the endoplasmic reticulum, while GPI anchor is required for anchoring AGPs to the plasma membrane. Therefore, these 191 proteins were further screened for the presence of a signal peptide and a GPI anchor. Fifty eight proteins longer than 90 amino acids and 24 proteins shorter than 90 amino acids were identified as they contain both the signal peptide and the GPI anchor (Table 1). By eliminating the proteins contained other domains such as fascilin-like domain and early nodulin-like domain, 22 classical AGPs, 24 AG peptides, and three lys-rich AGPs were identified as “high confidence” AGPs (Tables 1, 2). Additionally, the orthologous genes of all the annotated classical AGPs, AG peptides and lys-rich AGPs in *A. thaliana* were screened in the proteome of *B. rapa*. With this approach, 15 additional AGPs have been revealed as AGP candidates, including 11 classical AGPs, and four AG peptides (Table 2).

**Table 2:** **Identification, characterization and classification of putative AGP encoding genes in the *Brassica rapa* genome**.

**Subfamily**	***Arabidopsis thaliana***			***Brassica rapa***									
	**Gene name**	**Gene ID**	**Block**	**Gene name^a^**	**Gene ID**	**PAST (%)**	**SP^b^**	**GPI^c^**	**AP/PA/SP/TP repeats**	**Amino acids**	**Chromosome**	**Sub-genome**	**Block**
Classical	*AtAGP1*	At5g64310	X	***BrAGP1.1***	Bra024284	59	Yes	Yes	10/16/20/4	124	A06	LF	X
				***BrAGP1.2***	Bra031924	56	Yes	Yes	8/14/24/2	130	A02	MF1	X
	*AtAGP2*	At2g22470	I	***BrAGP2.1***	Bra038521	62	Yes	Yes	14/6/16/4	132	A09	LF	I
				***BrAGP2.2***	Bra030234	61	Yes	Yes	16/10/14/4	132	A04	MF1	I
	*AtAGP3*	At4g40090	U	***BrAGP3.1***	Bra011806	61	Yes	Yes	14/14/18/8	143	A01	LF	U
				***BrAGP3.2***	Bra010639	60	Yes	Yes	14/12/18/10	143	A08	MF2	U
	*AtAGP4*	At5g10430	R	***BrAGP4.1***	Bra009032	71	Yes	Yes	18/24/6/20	143	A10	LF	R
				***BrAGP4.2***	Bra006060	72	Yes	Yes	14/20/8/18	141	A03	MF1	R
				***BrAGP4.3***	Bra028587	71	Yes	Yes	20/20/6/16	139	A02	MF2	R
	*AtAGP5*	At1g35230	B										
	*AtAGP6*	At5g14380	R	***BrAGP6***	Bra008762	66	Yes	Yes	14/8/14/6	152	A10	LF	R
	*AtAGP7*	At5g65390	X										
	*AtAGP9*	At2g14890	H	***BrAGP9.1***	Bra013116	70	Yes	Yes	14/22/14/18	179	A03	MF2	H
				***BrAGP9.2***	Bra039829	67	Yes	Yes	14/18/16/16	166	Scaffold000178		
	*AtAGP10*	At4g09030	P	*BrAGP10.1*	Bra038390	62	Yes	No	6/2/8/6	81	A09	LF	P
				***BrAGP10.2***	Bra000670	65	Yes	Yes	12/8/12/10	126	A03	MF1	P
	*AtAGP11*	At3g01700	H	***BrAGP11.1***	Bra000995	59	Yes	Yes	16/4/8/4	136	A03	MF2	H
				***BrAGP11.2***	Bra040548	63	Yes	Yes	18/6/10/4	138	Scaffold000217		
	*AtAGP25*	At5g18690	R	***BrAGP25***	Bra002180	50	Yes	Yes	8/4/14/2	135	A10	LF	R
	*AtAGP26*	At2g47930	J	***BrAGP26***	Bra021448	52	Yes	Yes	4/2/20/2	140	A04	MF1	J
	*AtAGP27*	At3g06360	F	***BrAGP27***	Bra040224	55	Yes	Yes	8/6/8/0	126	A01	MF1	F
	*AtAGP50*	At1g24520	B	*BrAGP50.1*	Bra032796	46	Yes	Yes	4/0/8/2	119	A09	LF	B
				*BrAGP50.2*	Bra010966	48	Yes	Yes	6/2/8/2	117	A08	MF1	B
				*BrAGP50.3*	Bra012505	47	Yes	Yes	4/2/10/0	119	A07	MF2	B
	*AtAGP51*	At1g31250	B										
	*AtAGP52*	At1g63530	D	*BrAGP52*	Bra027649	46	No	No	8/24/16/24	420	A09	MF2	D
	*AtAGP53*	At1g63540	D	*BrAGP53*	Bra027648	46	Yes	No	12/16/14/32	618	A09	MF2	D
	*AtAGP54*	At2g28440	I	***BrAGP54.1***	Bra011948	53	Yes	Yes	12/18/54/2	234	A07	LF	I
				*BrAGP54.2*	Bra035700	44	Yes	Yes	2/8/24/4	186	A04	MF1	I
	*AtAGP55*	At2g45000	J	*BrAGP55*	Bra004882	59	No	Yes	46/38/26/22	784	A05	LF	J
	*AtAGP56*	At3g22070	F										
	*AtAGP57*	At3g45230	M	*BrAGP57*	Bra038294	43	Yes	Yes	8/8/24/2	168	A06	LF	M
	*AtAGP58*	At4g16980	U	*BrAGP58.1*	Bra040103	56	No	No	8/4/16/8	135	A01	LF	U
				*BrAGP58.2*	Bra021074	58	No	Yes	12/2/16/4	130	A08	MF2	U
				***BrAGP59***	Bra028633	53	Yes	Yes	6/4/14/6	115	A02	MF2	R
				***BrAGP60***	Bra035584	60	Yes	Yes	24/24/50/18	339	A02	MF2	W
				***BrAGP61***	Bra036401	70	Yes	Yes	14/16/18/14	171	A07	LF	H
AG-peptide	*AtAGP12*	At3g13520	F	***BrAGP12.1***	Bra027425	44	Yes	Yes	2/4/4/0	61	A05	LF	F
				***BrAGP12.2***	Bra039397	47	Yes	Yes	2/4/4/0	62	Scaffold000164		
	*AtAGP13*	At4g26320	U	***BrAGP13.1***	Bra026448	46	Yes	Yes	4/4/2/0	59	A01	LF	U
				***BrAGP13.2***	Bra019114	42	Yes	Yes	4/2/2/0	59	A03	MF1	U
	*AtAGP14*	At5g56540	W	***BrAGP14***	Bra002808	43	Yes	Yes	4/2/2/0	60	A10	LF	W
	*AtAGP15*	At5g11740	R	***BrAGP15.1***	Bra008940	53	Yes	Yes	4/2/4/0	64	A10	LF	R
				***BrAGP15.2***	Bra006115	53	Yes	Yes	2/2/6/0	64	A03	MF1	R
				***BrAGP15.3***	Bra023339	52	Yes	Yes	4/2/4/0	64	A02	MF2	R
	*AtAGP16*	At2g46330	J	***BrAGP16.1***	Bra004546	42	Yes	Yes	6/4/0/0	73	A05	LF	J
				***BrAGP16.2***	Bra000419	42	Yes	Yes	6/4/0/0	73	A03	MF2	J
	*AtAGP20*	At3g61640	N	***BrAGP20***	Bra014427	40	Yes	Yes	4/2/4/0	68	A04	MF1	N
	*AtAGP21*	At1g55330	C	***BrAGP21.1***	Bra037993	46	Yes	Yes	4/4/2/0	59	A06	LF	C
				***BrAGP21.2***	Bra011914	46	Yes	Yes	4/4/2/0	59	A07	MF2	C
				***BrAGP21.3***	Bra030868	47	Yes	Yes	4/4/2/0	58	A08	MF1	C
	*AtAGP22*	At5g53250	W	***BrAGP22.1***	Bra003071	38	Yes	Yes	4/4/4/0	63	A10	LF	W
				***BrAGP22.2***	Bra029086	38	Yes	Yes	4/4/4/0	63	A03	MF1	W
				***BrAGP22.3***	Bra022641	38	Yes	Yes	4/4/4/0	63	A02	MF2	W
	*AtAGP23*	At3g57690	N	*BrAGP23.1*	Bra007349	46	No	Yes	4/6/0/0	61	A09	LF	N
				*BrAGP23.2*	Bra014611	44	No	Yes	4/6/0/0	61	A04	MF1	N
				***BrAGP23.3***	Bra003296	43	Yes	Yes	4/6/0/0	61	A07	MF2	N
	*AtAGP24*	At5g40730	S	***BrAGP24***	Bra025551	37	Yes	Yes	4/6/2/0	67	A04	LF	S
	*AtAGP40*	At3g20865	F	***BrAGP40.1***	Bra031236	45	Yes	Yes	4/4/2/0	64	A05	LF	F
				***BrAGP40.2***	Bra023919	47	Yes	Yes	4/4/2/0	64	A01	MF1	F
	*AtAGP41*	At5g24105	Q										
	*AtAGP42*	At1g51915	C	*BrAGP42*	Bra030409	30	Yes	No	0/2/2/0	77	A05	MF2	C
	*AtAGP43*	At2g41905	J	***BrAGP43.1***	Bra016902	44	Yes	Yes	4/6/0/0	61	A04	MF1	J
				***BrAGP43.2***	Bra000244	44	Yes	Yes	4/6/0/0	61	A03	MF2	J
	*AtAGP44*	At3g01730	F	***BrAGP44***	Bra000992	39	Yes	Yes	4/2/4/0	90	A03	MF2	F
	*AtAGP45*	At5g12880	R	*BrAGP45*	Bra008874	46	Yes	No	2/0/4/0	76	A10	LF	R
Lys-rich	*AtAGP17*	At2g23130	I	***BrAGP17***	Bra039184	68	Yes	Yes	44/42/56/12	305	A09	LF	I
	*AtAGP18*	At4g37450	U	***BrAGP18.1***	Bra011767	62	Yes	Yes	20/14/26/4	178	A01	LF	U
				***BrAGP18.2***	Bra017823	65	Yes	Yes	30/30/28/10	202	A03	MF1	U
	*AtAGP19*	At1g68725	E										

a Boldface indicates a protein that is treated as “high confidence” AGPs;

b Signal peptide;

c *GPI was predicted both by big-PI Plant Predictor and PSORT Prediction*.

All AGPs in *B. rapa* were named after their orthologous proteins in *A. thaliana*. Bra038390 (81 amino acids in length) was identified as the ortholog of a classical AGP—AtAGP10. Hence, for convenience, Bra038390 was regarded as a classical AGP but not as an AG peptide. Moreover, sequence homology analysis did not result in any orthologous proteins in the annotated AtAGPs for three BrAGPs, Bra028633, Bra035584, and Bra036401, so they were designated as BrAGP59, BrAGP60, and BrAGP61, respectively. Overall, apart from the chimeric proteins, 64 AGPs were detected in *B. rapa*, including 33 classical AGPs, 28 AG peptides and three lys-rich AGPs (Table 2).

### Retained proportion analysis

**Figure 1:**
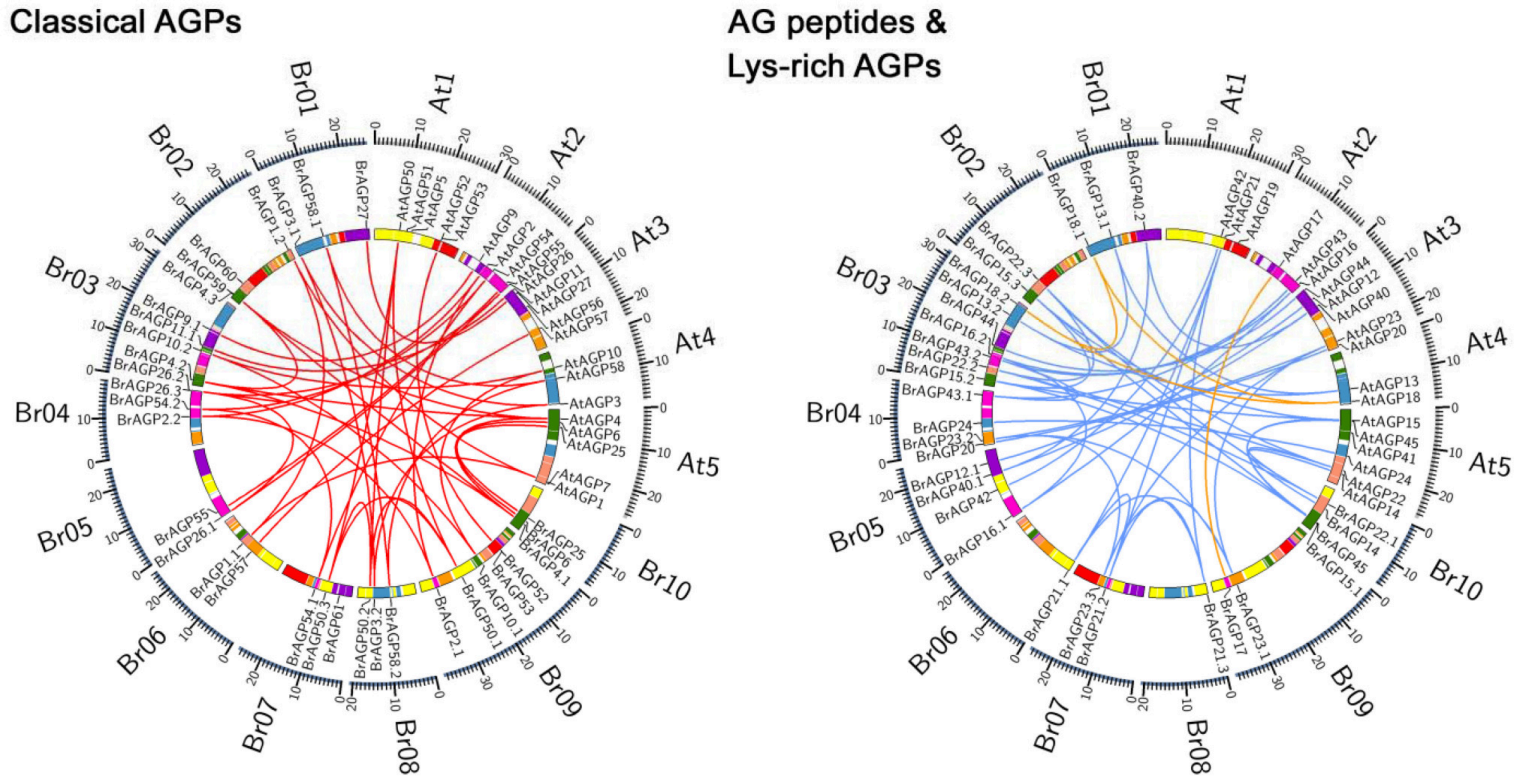
**Syntenic analysis of *AGPs* in *Brassica rapa* and *Arabidopsis thaliana***. The syntenic relationships between *AGPs* in *B. rapa* and *A. thaliana* are displayed according to the syntenic gene search function in Brassica database (BRAD). Classical AGPs encoding genes are linked in red line, AG peptides encoding genes are linked in blue lines and lys-rich AGPs encoding genes are linked in yellow lines.

Most of the *BrAGPs* were distributed on chromosomes, with the exception of *BrAGP9.2, BrAGP11.2*, and *BrAGP12.2*, which were located on scaffolds. On average, the chromosomes contained four to nine *BrAGPs*, with chromosome A06 harboring only three and chromosome A03 having up to 11. The 24 conserved collinear blocks in ancestral karyotype were used to identify the syntenic relationship between *BrAGPs* and their corresponding orthologous genes in *A. thaliana*. The result illustrated that all *BrAGPs* are located in the same blocks as their *A. thaliana* orthologous genes (Figure 1), suggesting that the expansion of *AGP* gene family in *B. rapa* was depended on the WGT event.

**Figure 2:**
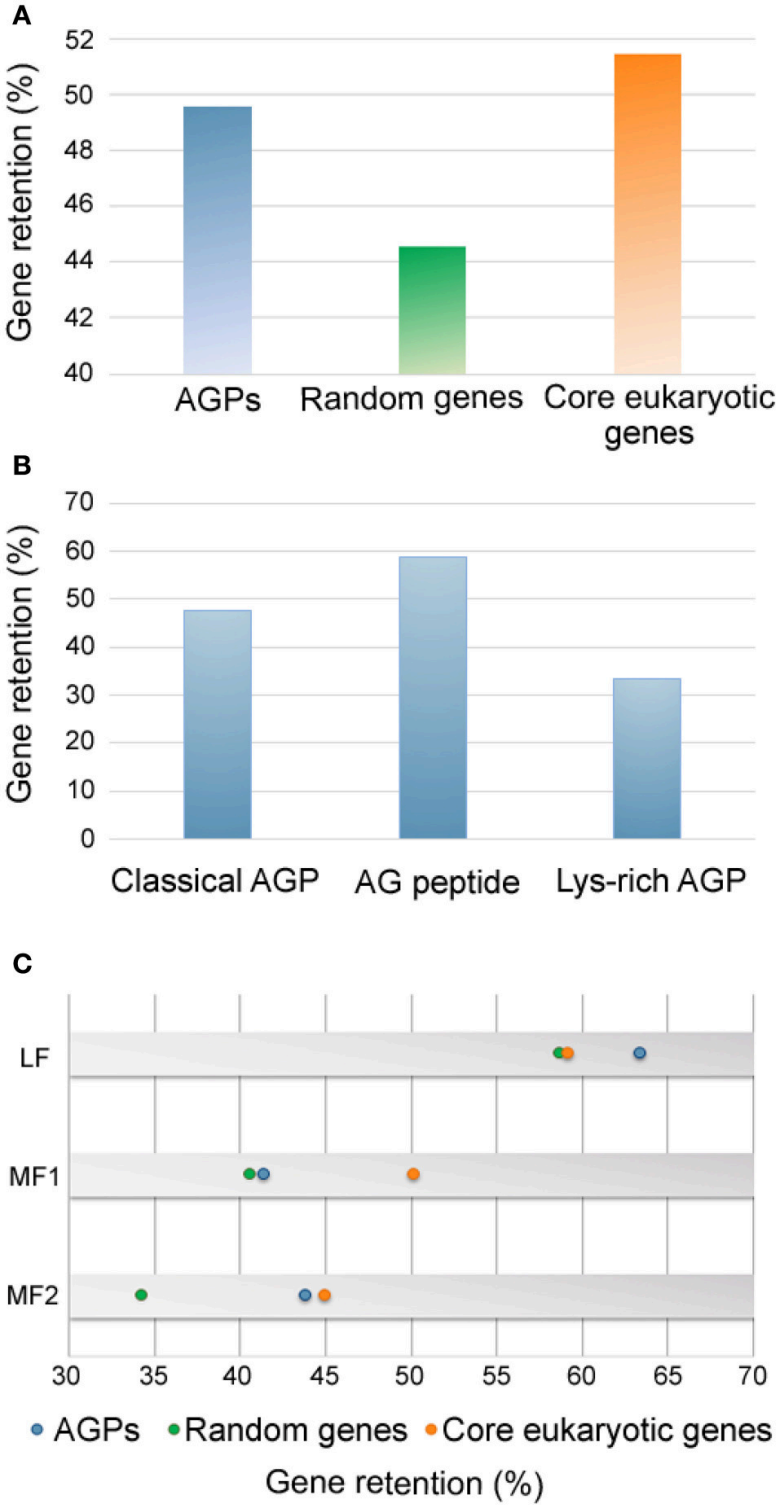
**The retained proportion of *AGPs* in *Brassica rapa*. (A)** The retained proportions of *AGPs* (blue), random genes (green) and core eukaryotic genes (orange) in *B. rapa*. **(B)** The retained proportions of classical AGP, AG peptides and lys-rich AGPs encoding genes. **(C)** The retained proportions of AGP encoding genes (blue), random genes (green) and core eukaryotic genes (orange) in different subgenomes of *B. rapa*.

No orthologous genes of *AtAGP5, AtAGP7, AtAGP19, AtAGP41, AtAGP51*, and *AtAGP56* were identified in *B. rapa*. To investigate whether these *AtAGPs* were newly acquired in *A. thaliana* or were lost in *B. rapa*, their orthologous genes in other sequenced *Brassica* species were analyzed. *Brassica* species can be classified into three lineages by spectrum analysis, and most sequenced species were concentrated on Lineage I and Lineage II. Specifically, *A. thaliana, A. lyrata, C. rubella*, and *C. sativa* belonged to Lineage I, while *B. rapa, B. napus, B. oleracea, T. halophile, T. salsuginea, S. irio*, and *S. parvula* were in Lineage II (Koch and German, 2013). Analysis of these orthologous genes illustrated that *AtAGP5, AtAGP7, AtAGP19, AtAGP41*, and *AtAGP56* exist not only in the species belonging to Lineage I, but also in the species belonging to Lineage II as well as in the primitive *Brassica* species, *A. arabicum*. This finding indicated that these genes may be lost in *B. rapa* (Supplementary data 1: Figure S1). The orthologs of *AtAGP51* could only be detected in species in Lineage I, indicating that this gene arose after the separation of *A. thaliana* and *B. rapa*. Based on this result, the retained proportion of *AGPs* in *B. rapa* was calculated. The *AGP* gene family had a retained proportion of 50%, which was much higher than that of randomly selected genes (45%) and similar to that of core eukaryotic genes (52%) (Figure 2A). Particularly, classical AGPs, AG peptides, and lys-rich AGPs had a proportion of 48, 58, and 33%, respectively (Figure 2B).

As illustrated in the whole genome level, *BrAGPs* also displayed differentially-retained proportions among the three subgenomes, namely least fractionated subgenome (LF), medium fractionated subgenome (MF1) and most fractionated subgenome (MF2) of *B. rapa* (Figure 2C). The *AGP* gene family contained more genes of LF subgenome (63%) than those of random selected genes (59%) and core eukaryotic genes (59%), while it had similar proportion of core eukaryotic genes in the MF1 and MF2 subgenomes for *AGP* genes and core eukaryotic genes, respectively. This indicated that the highly retained proportion of *AGPs* is mainly attributed to the gene reservation in LF.

### Phylogenetic and protein structure analysis

**Figure 3:**
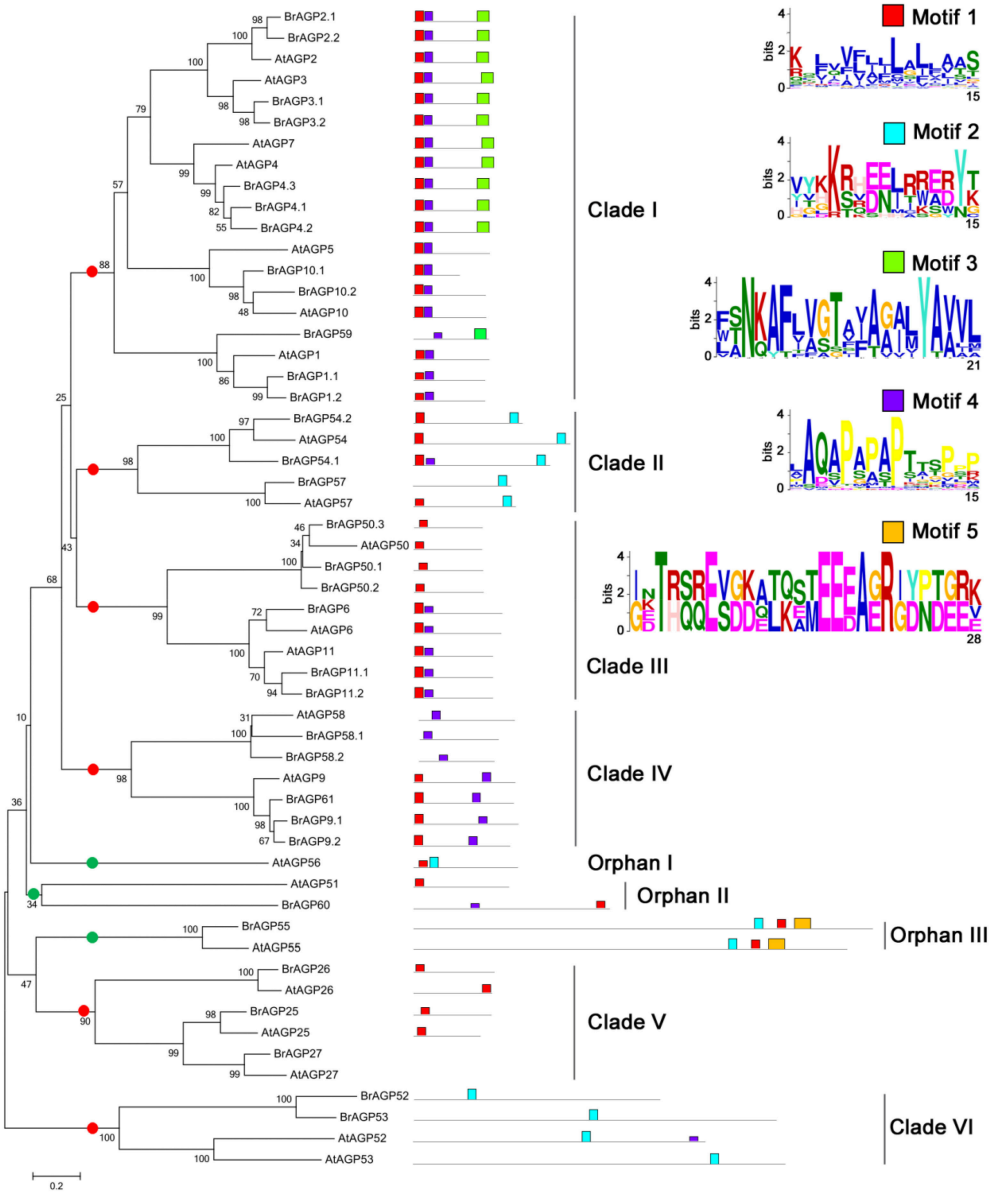
**Phylogenetic and motif analysis of classical AGPs in *Brassica rapa* and *Arabidopsis thaliana***. Phylogenetic tree of classical AGPs was constructed in whole protein sequences using neighbor-joining with 1,000 bootstrap in MEGA 6.0. Motif analysis was performed by MEME software. Red points are for genes in clades and green points are for orphan genes.

Full length protein sequences of all identified *BrAGPs* and their orthologs in *A. thaliana* were used to construct phylogenetic trees. The topologies of NJ and ML trees were mainly consistent, thus only the NJ trees are presented here (Figure 3). The 55 classical AGPs in *B. rapa* and *A. thaliana* were classified into six clades and three orphan genes with high bootstrap support (Figure 3). Motif analysis showed that the AGPs in Clade I mainly have two types of structural features. AGP2, AGP3, AGP4, and AGP7 contained motif 1, motif 4 and motif 3, while AGP1, AGP5, and AGP10 included motif 1 and motif 4. Clade II was constituted of AGP54 and AGP57 with motif 1 and motif 2. Clade III had three members, AGP6, AGP11, and AGP50, in which AGP6 and AGP11 had motif 1 and motif 4, similar to some of the members in Clade I, while AGP50 only had motif 1. AGP9, AGP58, and AGP62 belonged to Clade IV with AGP58 having only motif 4, while AGP9 and AGP62 having motif 1 and motif 4. Clade V was composed of AGP25, AGP26, AGP27, which only hold motif 1. Tandem gene pair, AGP52 and AGP53 contained motif 4. Phylogenetic analysis indicated that the tandem repeat event occurred after the separation of *B. rapa* and *A. thaliana*, as the AGPs belonging to *B. rapa* had a closer resemblance to each other than to the orthologs in *A. thaliana*.

**Figure 4:**
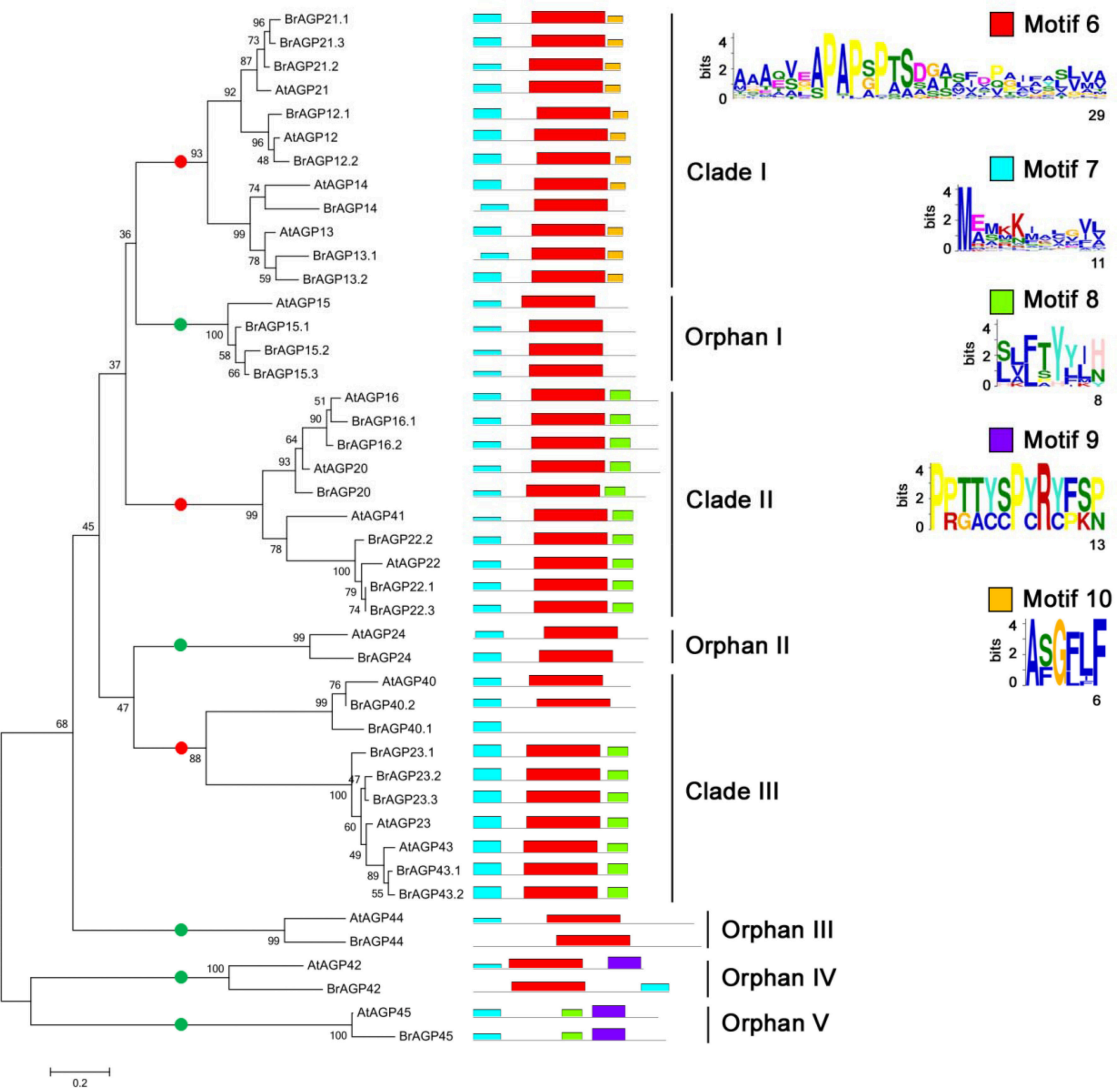
**Phylogenetic and motif analysis of AG peptides in *Brassica rapa* and *Arabidopsis thaliana***. Phylogenetic tree of AG peptides was constructed in whole protein sequences using neighbor-joining with 1,000 bootstrap in MEGA 6.0. Motif analysis was performed by MEME software. Red points are for genes in clades and green points are for orphan genes.

Phylogenetic analysis of AG peptides led to the division of three clades and five orphan genes, and almost all AG peptides had motif 6 and motif 7 (Figure 4). Four members of Clade I, AGP12, AGP13, AGP14, and AGP21, had motif 10 in addition to motif 6 and motif 7. AGP16, AGP20, AGP22, and AGP41 in Clade II all contained each motif 6, motif 7 and motif 8. Clade III included AGP23 and AGP43, which all had motif 8.

### Estimation of the molecular evolutionary rates of AGPs

**Figure 5:**
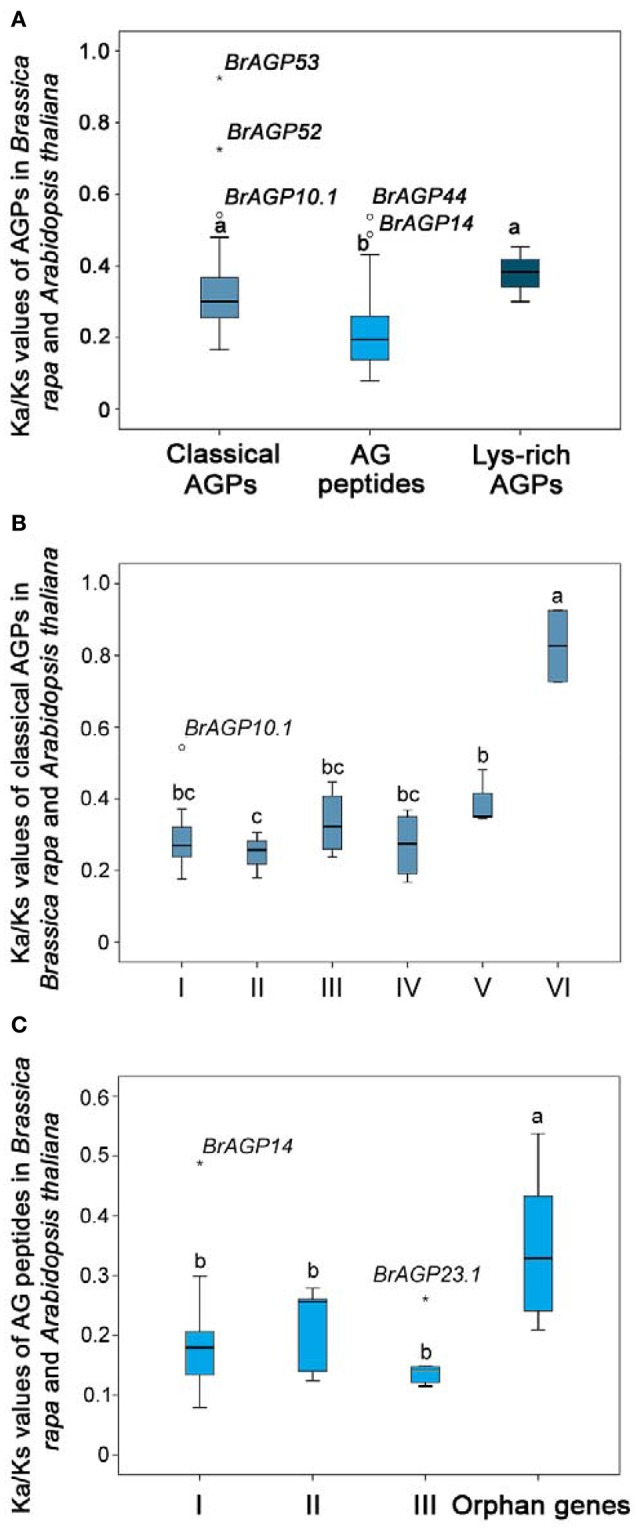
**Molecular evolutionary rate of *AGPs* in *Brassica rapa*. (A)** Rates of molecular evolution of classical AGPs, AG peptides, and lys-rich AGPs encoding genes were estimated using YN86 in PAML software by calculating the Ka and Ks values between *AGPs* in *B. rapa* and their orthologous genes in *Arabidopsis thaliana*. **(B)** Rates of molecular evolution in each clades of classical AGPs encoding genes. **(C)** Rates of molecular evolution in each clade of AG peptides encoding genes. Different letters indicate statistical significance (*P* < 0.05) as determined by a one-way ANOVA test.

Estimation of molecular evolution rates forms an important basis for our understanding of the evolution processes in *B. rapa*. YN86 module was used for estimating the molecular evolutionary rates of the orthologous gene pairs among *AGPs* (Supplementary data 1: Table S3). All three types of *AGPs* encoding genes were evolved at a Ka/Ks value lower than 1, indicating that they all have evolved through purifying selection (Figure 5A). Classical AGPs showed an average Ka/Ks value of 0.30, AG peptides exhibited and Lys-rich AGPs were evolved at an average Ka/Ks value of 0.20 and 0.38, respectively. Statistical analysis illustrated that classical AGPs have evolved faster than AG peptides, but there is no significant differences existed between classical AGPs and lys-rich AGPs. Furthermore, the average molecular evolutionary rates of each clade were compared. In classical AGPs, Clade VI had a significantly higher Ka/Ks value than the other five clades (Figure 5B). In AG peptides, Clade I, Clade II, and Clade III shared a similar molecular evolutionary rate, while the orphan genes evolved at a faster rate of evolution (Figure 5C).

### Differential expression of *BrAGPs*

**Figure 6:**
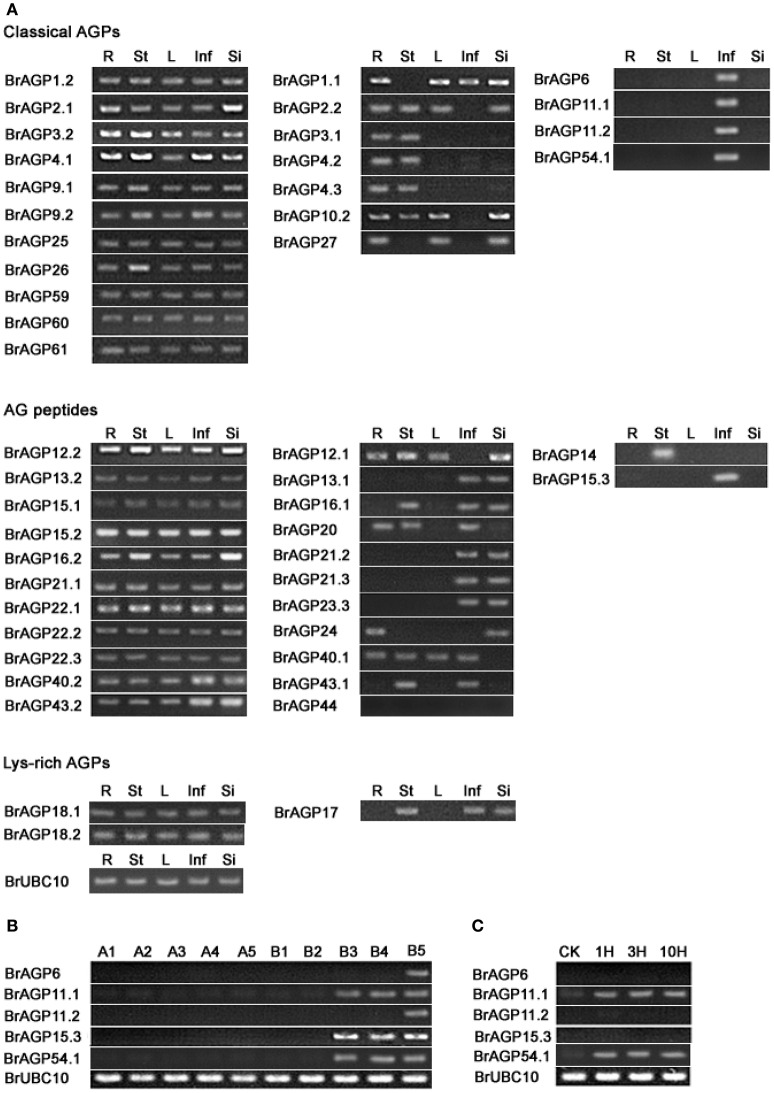
**Differential expression of *AGPs* in *Brassica rapa*. (A)** Expression levels of *AGPs* in root (R), stem (St), leaf (L), inflorescence (Inf), and silique (Si) are detected by RT-PCR. **(B)** Expression of inflorescence-specific *AGPs* in flower buds of fertile line “*Bcajh97-01B*” and sterile line “*Bcajh97-01A*.” B1–B5 are five stages of flower buds in “*Bcajh97-01B*,” which are corresponding to five developmental stages of pollen; A1–A5 indicate five stages of flower buds in “*Bcajh97-01A*,” which have the similar longitudinal diameter to the corresponding flower buds in “*Bcajh97-01B*.” **(C)** Expression of inflorescence-specific *AGPs* in unpollinated pistils (CK) and pollinated pistils at 1 h after pollination (1 HAP), 3 HAP, and 10 HAP.

Half of the classical *BrAGPs* showed expression in all of the tissues detected, including root, stem, leaf, inflorescence and silique. Seven *AGPs* were expressed in more than one but not all tissues. *BrAGP6, BrAGP11.1, BrAGP11.2*, and *BrAGP54.1* expression was restricted in inflorescence (Figure 6A). Eleven of the 24 AG peptides encoding genes were expressed in all tissues with *BrAGP12.2, BrAGP13.2, BrAGP15.1, BrAGP15.2, BrAGP22.1*, and *BrAGP22.2* displaying similar expression levels among different tissues. Half of the genes encoding AG peptides were expressed in different tissues, of which *BrAGP15.3* was inflorescence-specific and *BrAGP14* was expressed exclusively in the stem. The expression of *BrAGP44* was not detected in any of the tissues (Figure 6A).

Among the three lys-rich AGPs encoding genes, *BrAGP18.1* and *BrAGP18.2* were expressed in all tissues, while *BrAGP17* was only detectable in stem, inflorescence and silique (Figure 6A). Interestingly, most paralogous gene pairs displayed different expression patterns. Specifically, from the classical AGPs group, the gene pairs of *BrAGP1.1* and *BrAGP1.2, BrAGP2.1* and *BrAGP2.2, BrAGP3.1* and *BrAGP3.2, BrAGP4.1* and *BrAGP4.2/BrAGP4.3* had differential expression patterns. Likewise, the paralogous gene pairs of *BrAGP12.1* and *BrAGP12.2, BrAGP13.1* and *BrAGP13.2, BrAGP15.1, BrAGP15.2* and *BrAGP15.3, BrAGP16.1* and *BrAGP16.2, BrAGP21.1, BrAGP21.2* and *BrAGP21.3, BrAGP40.1* and *BrAGP40.2, BrAGP43.1* and *BrAGP43.2*, encoding for AG peptides, showed differential expression patterns.

For the five inflorescence-specific genes, including four classical AGPs and one AG peptide encoding genes, their expression were further analyzed among different developmental stages of pollen in subdivided flower buds from the “*Bcajh97-01A/B*”GMS line (Figure 6B). These five *BrAGPs* were expressed in the fertile but not in sterile flower buds. *BrAGP6* and *BrAGP11.2* were only expressed at Stage I which corresponds to the mature pollen stage, while *BrAGP11.1, BrAGP15.3*, and *BrAGP54.1* were detected at Stage III to V, which means that they are expressed throughout the uninucleate pollen stage to the mature pollen stage. Since some genes expressed in mature pollen usually continue to be expressed in germinating pollen and pollen tubes, the expression levels of the five *BrAGPs* were investigated in pistils at three different time points after pollination in a fertile line. *BrAGP11.1* and *BrAGP54.1* were found to be expressed in pistils during the whole fertilization processes; however the other three genes were not detected in the pistils (Figure 6C).

### Effects of exogenous GA, MeJA, and ABA on the expression of *BrAGPs* in the leaf

Phytohormones serve as key signals for the plants to respond to environmental stimuli (Wania et al., 2016). In order to investigate whether *BrAGPs* were involved in phytohormone responses, we chose 15 classical AGPs, 13 AG peptides and two lys-rich AGPs, which according to our qRT-PCR displayed expression in the leaf. Three vital phytohormones, ABA, GA, and MeJA, involved in abiotic and biotic stress resistance, were used in this study to investigate the leaf *BrAGPs* expression in response to phytohormone treatments.

**Figure 7:**
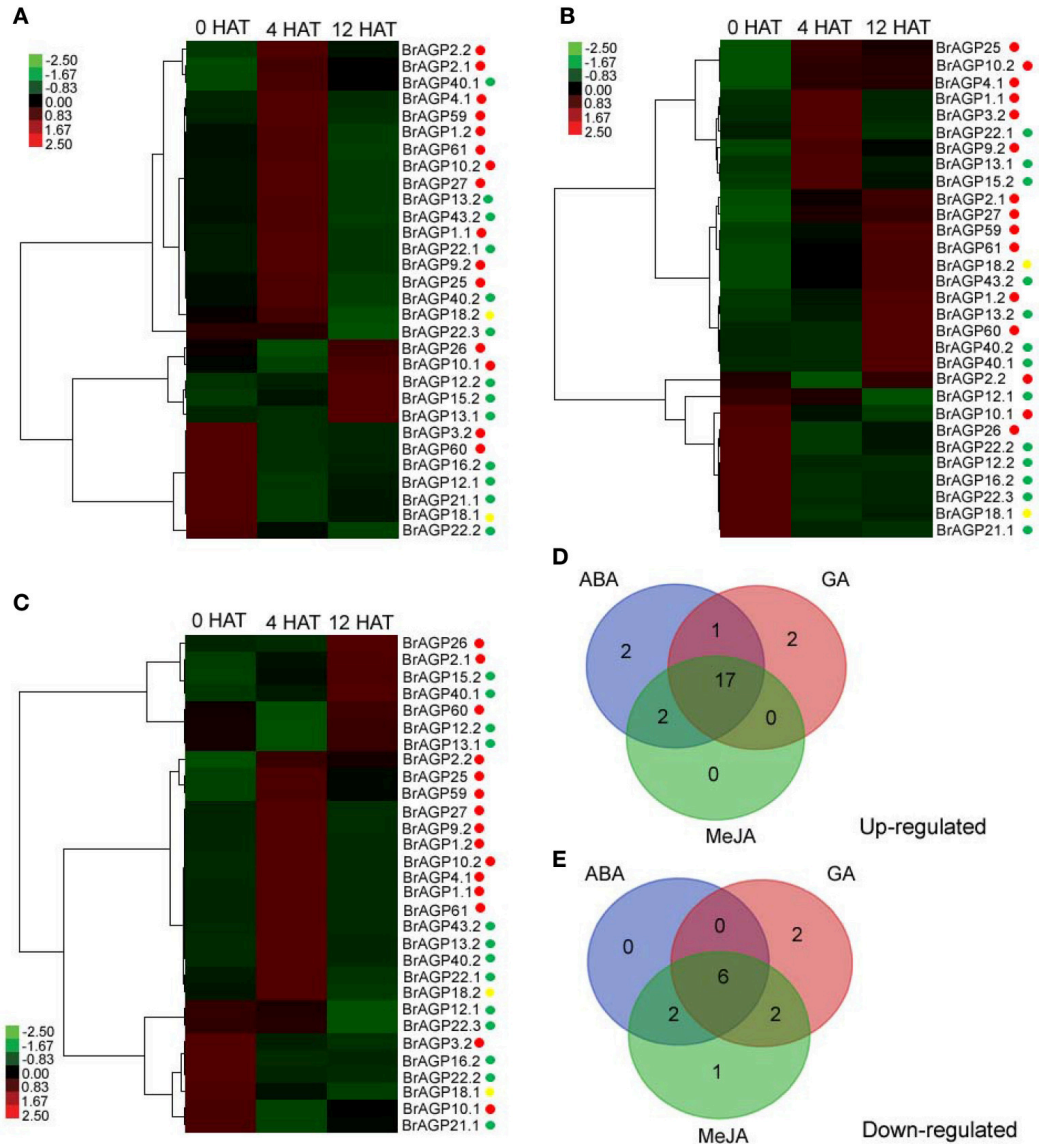
**Responses of *AGPs* in *Brassica rapa* to exogenous phytohormones treatments**. Responses of leaf-expressed *AGPs* to exogenous ABA **(A)**, GA **(B)**, and MeJA **(C)** are detected by qRT-PCR at 0 h after treatment (HAT), 4 HAT, and 12 HAT. The gene expression under treatments were normalized to the corresponding distilled water treatment and calculated through 2^−ΔΔCt^ method, displayed in heatmap and clustered hierarchically by Cluster 3.0. Red points are for classical AGPs encoding genes, green points are for AG peptides encoding genes, and yellow points are for lys-rich AGPs encoding genes. Venn diagrams of up-regulated *AGPs* **(D)** and down-regulated *AGPs* **(E)** after the treatment of exogenous ABA, GA, and MeJA.

Following ABA treatment, 22 *BrAGPs* were up-regulated, including 13 classical AGPs, eight AG peptides and one lys-rich AGPs encoding genes (Figure 7A). Expression of 11 classical AGPs, four AG peptides, and a lys-rich AGPs encoding genes were markedly increased at 4 HAT and then recovered at 12 HAT; whereas, the expression levels of two classical AGPs and three AG peptides encoding genes were increased at 12 HAT. Eight *BrAGPs*, including two classical AGPs, five AG peptides, and one lys-rich AGPs encoding genes were down-regulated upon ABA treatment. Their expression levels decreased mainly at 4 HAT and didn't recover at 12 HAT, while *BrAGP22.3* decreased at 12 HAT. Except *BrAGP2.1* and *BrAGP2.2* that shared similar responses, most paralogous gene pairs displayed different expression patterns after ABA treatment.

Up-regulation of 12 classical AGPs, seven AG peptides, and one lys-rich AGPs genes was observed after GA treatment (Figure 7B). The expression levels of three classical AGPs encoding genes were increased at 4 HAT and subsequently at 12 HAT. Three classical AGPs and three AG peptides encoding genes were also up-regulated at 4 HAT but their transcript levels dropped back at 12 HAT. There was no effect on the expression of genes encoding for six classical AGPs, four AG peptides, and one lys-rich AGP, until 12 HAT. In contrast, the expression of encoding genes for three classical AGPs, six AG peptides, and one lys-rich AGP was down-regulated. The expression level of *BrAGP2.2* decreased at 4 HAT and recovered at 12 HAT, while *BrAGP12.1* was down-regulated at 12 HAT. The remaining genes were down-regulated at 4 HAT and sustained low expression levels at 12 HAT. Although the expression of most paralogous gene pairs was different, the response pattern between *BrAGP40.1* and *BrAGP40.2*, and between *BrAGP22.2* and *BrAGP22.3*, was similar (Figure 7B).

Nineteen *BrAGPs* were up-regulated in MeJA treatment. Two classical AGPs and two AG peptides encoding genes were induced at 12 HAT, while 10 classical AGPs, four AG peptides and one lys-rich AGPs encoding genes, were up-regulated at 4 HAT and recovered at 12 HAT (Figure 7C). In addition, 11 AGPs were down-regulated, following MeJA treatment. One classical AGP and two AG peptides were down-regulated at 4 HAT and recovered at 12 HAT, while the expression levels of the encoding genes for two classical AGPs, five AG peptides, and one lys-rich AGP decreased at 4 HAT and didn't recover at 12 HAT. *BrAGP1.1* and *BrAGP1.2* showed the same response to ABA, but the rest of the paralogous gene pairs displayed different expression patterns.

Interestingly, the ABA-, GA-, and MeJA-induced expression of *BrAGPs* were largely overlapped. Seventeen *BrAGPs* were up-regulated by all three phytohormones, including genes encoding 10 classical AGPs, six AG peptides, and one lys-rich AGPs. In addition, *BrAGP2.2* and *BrAGP26* were up-regulated by both ABA and MeJA, and *BrAGP13.1* was up-regulated both by ABA and GA (Figure 7D). Five AG peptides encoding genes and one lys-rich AGP encoding gene were down-regulated by all three phytohormones. However, *BrAGP3.2* and *BrAGP60* were solely down-regulated by ABA and MeJA, the expression levels of *BrAGP10.1* and *BrAGP12.2* decreased only in response to GA and MeJA, respectively (Figure 7E).

Comparison of the gene expression induction kinetics of different hormones revealed that the overlap between the response to ABA and MeJA is much bigger than that between ABA and GA, or MeJA and GA (Figure 7). In general, most of the transcriptional response of *BrAGPs* to ABA and MeJA treatments was rapid and transient with expression changes detected at 4 HAT, while these of GA were relatively slow (expression changes were not detectable until 12 HAT).

## Discussion

### The high retained proportions of AGPs in *B. rapa* may be attributed to subfunctionalization

AGPs are characterized by the repetitive nature of their protein backbones with less than 40% similarity between them (Schultz et al., 2002). The low similarity makes it difficult to be identified using the basic local alignment search tool (BLAST) or profile hidden Markov model (HMM) methods. “Hyp contiguity hypothesis” has provided a guidance to identify AGPs by Perl scripts (Kieliszewski and Lamport, 1994). However, it may lead to variable results if different programs and standards are employed (Schultz et al., 2002; Showalter et al., 2010). Here, based on the threshold values set in Showalter et al. (2010), a whole genome wide screening of classical AGPs, AG peptides, and lys-rich AGPs was performed in *B. rapa*, resulting in the identification of 64 *BrAGPs* including 49 with high confidence.

*B. rapa* contains more *AGPs* than *A. thaliana*, especially classical AGPs and AG peptides that is mainly attributed to the WGT event. However, we could not identify orthologs for three *BrAGPs* in *A. thaliana*, and orthologs for six *AtAGPs* in *B. rapa*. Ortholog comparison with closely related *Brassica* species revealed that five *AtAGPs* are lost in *B. rapa*, while one *AtAGP* and three *BrAGPs* were independently evolved after the separation of *A. thaliana* and *B. rapa*. These results indicate that besides the WGT event, other processes, for instance, dosage effect, might have also contributed to the evolution of the AGP family. In this study we have shown that classical AGPs, AG peptides and lys-rich AGPs are highly represented in the genome of *B. rapa*. Similar results were also reported by two previous studies where 33 FLAs and 52 ENODLs were identified in *B. rapa*. The corresponding retained proportions for these two types of chimeric AGPs were described to be 52 and 67%, respectively (Jun and Xiaoming, 2012; Li et al., 2013).

According to previous study, the over retained genes or gene families were always dosage sensitive or usually undergone subfunctionalization and/or neofunctionalization (Kim et al., 2014). In our investigation of the expression of *BrAGPs*, we revealed that 4/5 “high confidence” classical AGP and 6/7 AG peptide paralogous gene pairs had distinct expression patterns in different tissues. Such results indicated that the high retained proportions of classical AGPs and AG peptides are probably due to subfunctionalization and/or neofunctionalization, but not due to the dosage effect, which may yield increased expression of a gene (Edger and Pires, 2009). However, the result of molecular evolutionary rate analysis indicated that these AGPs were all under purifying selection, illustrating that neofunctionalization may have hardly occurred in these proteins. We thus suggest that subfunctionalization is the most likely mechanism that facilitated the retention of duplicated genes in classical AGPs and AG peptides. Unfortunately, in the previous studies on FLAs and ENODLs, the paralogous gene pairs in *B. rapa* and *A. thaliana* were treated as one gene for their gene expression pattern (Jun and Xiaoming, 2012; Li et al., 2013). Therefore, we were unable to verify whether the high retained proportion of the chimeric AGPs are also depended on subfunctionalization. Relevant research work should be focused on this issue in the future.

### Implication of a subset of AGPs in sexual reproduction

Comprehensive studies on gene expression can provide useful information for predicting gene function. In this study, we first investigated the expression of *BrAGPs* in five different tissues and found tissue-specific and non-tissue specific genes. To identify candidate AGPs that may be involved in pollen development, those inflorescence-specific *BrAGPs* including four classical AGPs encoding gene *BrAGP6, BrAGP11.1, BrAGP11.2, BrAGP54.1*, and one AG peptide encoding gene *BrAGP15.3*, were further investigated for their in male fertile/sterile flower buds at five different developmental stages. All these genes were specifically expressed in fertile flower buds at the late developmental stages, indicating a role in pollen formation or pollen function. Some genes expressed in mature pollen usually continued to be expressed in germinating pollen and pollen tubes, showing that they are specifically involved in the regulation of the subsequent pollination and fertilization processes (Pina et al., 2005; Becker and Feijo, 2007; Wang et al., 2008). We also detected the expression of these genes in pistils at three different time points after pollination. *BrAGP11.1* and *BrAGP54.1* were shown to be expressed in pistils during the whole fertilization processes, which indicated that they might also function in subsequent sexual reproductive events. A previous study has demonstrated that *AtAGP6* and *AtAGP11* are the only two pollen-specific classical *AGP*s and are essential for pollen and pollen tube function in *A. thaliana* (Pereira et al., 2006; Levitin et al., 2008). Recent studies using qRT-PCR and *in situ* hybridization in our laboratory also revealed that the homologous gene *BrAGP6* (formerly named as *BcMF18*) was specifically expressed in pollen (unpublished data), and *BrAGP11.1* (or *BcMF8*) was expressed in the developing pollen and the pollen tube (Huang et al., 2008b; Lin et al., 2014). The functional disruption of *BrAGP6* by antisense RNA technology caused collapse of the pollen due to the absence of cellular content and nucleus, and failure of intine layer formation due to lack of cellulose deposition (unpublished data). The inhibition of *BrAGP11.1* resulted in slipper-shaped and bilaterally sunken pollen with abnormal intine development and aperture formation, an arrest of pollen germination and unstable pollen tube formation (Lin et al., 2014). Although the functions of *BrAGP15.3* and *BrAGP54.1* are not fully understood, their expression patterns are similar to the known pollen-related AGP genes. Therefore, we suggest that these two genes may play important roles in pollen development and/or fertilization. Our results also revealed that only a small number of *BrAGPs* was inflorescence-specific. This is in agreement with a previous expression analysis of the FLAs and ENODLs in *B. rapa*, which also showed that only two BrENODL genes and five FLA genes were specifically expressed in inflorescence (Jun and Xiaoming, 2012; Li et al., 2013). Similarly, in *A. thaliana*, only a subset of *AGP* genes is expressed in pollen grain and pollen tubes (Pereira et al., 2006). Therefore, we speculate that, despite of the diversity of AGPs, only a small number of AGPs are endowed with a specific and defined role in male sexual reproduction in *B. rapa* as those in *A. thaliana*.

### Novel roles of BrAGPs in phytohormone signaling

AGPs are also implicated in signal recognition and transduction (Suzuki et al., 2002; van Hengel and Roberts, 2003), although the exact mechanisms are still elusive. The mRNA level of *AtAGP31* decreases at 8 h after ABA, MeJA, and wounding treatment (Liu and Mehdy, 2007), while *AtAGP30* specifically responds to ABA treatment (van Hengel and Roberts, 2003). Moreover, a mutation of this gene showed suppression of the ABA-induced delay in germination and alteration of the expression of some ABA-regulated genes (van Hengel and Roberts, 2003). In rice, two classical AGPs, *OsAGP1*, and *OsAGP15* were significantly up-regulated under the ABA treatment, indicating that they may response to ABA (Ma and Zhao, 2010). Moreover, AGPs are also involved in response to GA, for example, AGPs are suggested to play important roles in GA-induced α-amylase production in barley aleurone cells (Suzuki et al., 2002). The ATH1 microarray analysis results (GEO accession number: GSE39384) also revealed that many AGP-encoding genes are regulated by ABA, GA, and MeJA in *A. thaliana* (Supplementary data 2: Table S4). A similar trend was observed for some homologous *AGPs* between *B. rapa* and *A. thaliana*. For example, *AGP1* and *AGP2* were up-regulated in *A. thaliana* after 3 h treated by ABA, while their orthologs in *B. rapa* were also up-regulated at 4 HAT by ABA (Figure 7). The conserved response to the phytohormone treatments in different plant species indicates that *AGPs* may be important functional genes for phytohormone signaling pathways. However, many homologous *BrAGP* genes show diverse change trends under phytohormone treatments with their *A. thaliana* homologs. This result demonstrated that the differentiation of the AGP genes might be intended to acclimatize the plant to specific environmental changes. In our study, 17 *BrAGPs* were up-regulated and six were down-regulated by all three types of phytohormones, ABA, GA, and MeJA treatments. These genes comprise the majority (approximately 76%) of *AGPs* which are expressed in the leaf of *B. rapa*. These findings indicate that quite a few AGPs might be directly associated or indirectly interact with the hormone signaling network.

The molecular mechanism by which AGPs are involved in phytohormone signaling remains to be elucidated. The relative rapid expression of *AGPs* under exogenous phytohormone treatments suggested that they may function in maintaining the organism's homeostasis rather than solely being structural components. In plants, sugar levels reflect the plant's physiological conditions and sugar signaling is closely associated with multiple phytohormone signaling cascades and abiotic stress signaling (Smeekens, 2000; Gibson, 2005; Rolland et al., 2006). Interestingly, sugar can induce ABA in the sugar-ABA signaling cascade (Laby et al., 2000). Changes in the expression of each *BrAGP* gene by exogenous ABA treatment indicates that the AGPs may participate in the signaling cascade by the dynamic release and uptake of glycosyl radicals. It was clear that there is complex cross-talk among plant hormones, which are important in plant growth regulation and defense responses (Kohli et al., 2013; Wania et al., 2016). In this study, we have shown that there is a large overlap of the *BrAGPs* induced or suppressed by ABA, GA, and MeJA treatments. It indicates that these BrAGPs might participate in different signaling pathways or in overlapping processes that are controlled by phytohormonal interactions.

## Conclusion

We identified, for the first time, 33 classical AGPs, 28 AG peptides, and three lys-rich AGPs in the genome of *B. rapa*. We also elucidated their genomic characteristics, protein structures, duplication status, molecular evolutionary rates and expression characteristics in different tissues as well as under phytohormone treatments. High retained proportions of AGPs were observed in *B. rapa*, which may be resulted from subfunctionalization. Similar expression patterns to the known pollen-related *BrAGPs*, were found in the remaining two genes with unknown function, *BrAGP15.3* and *BrAGP54.1*, indicating they may also be involved in pollen development and/or fertilization. Large numbers of *BrAGPs* displayed responses under the treatment of ABA, MeJA, and/or GA, suggesting that they may participate in the response to phytohormone signaling independently and/or cooperatively. In summary, this study has provided fundamental information for revealing the roles of the classical AGPs, AG peptides, and lys-rich AGPs, three important types of AGPs in *B. rapa*.

## Author contributions

TH performed the identification of AGPs, expression analysis, and participated in drafting the manuscript. HD performed the protein structure and evolution analysis, and participated in manuscript preparation. JCu, ML, SL, and JCa participated in phytohormone treatments, expression analysis, identification of AGPs, research design, respectively. LH conceived the project, designed research, and revised the manuscript. All authors read and approved the final manuscript.

### Conflict of interest statement

The authors declare that the research was conducted in the absence of any commercial or financial relationships that could be construed as a potential conflict of interest.

## Abbreviations

AGPs: Arabinogalactan proteins
ABA: abscisic acid
AK: ancestral karyotype
AtAGPs: *A. thaliana* AGPs
BLAST: the basic local alignment search tool
BRAD: Brassica database
BrAGPs: *B. rapa* AGPs
ENODL: eNod-like AGPs
EXTs: extensions
FLAs: fasciclin-like AGPs
GA: gibberellin
GPI: glycosylphosphatidylinositol
HAP: hour after pollination
HAT: hours after treatment
HMM: hidden Markov model
Ka: nonsynonymous substitution rate
Ks: synonymous substitution rate
MeJA: methyl jasmonate
MEME: Multiple Em for Motif Elicitation
ML: maximum likelihood
NJ: neighbor-joining
P/HRGPs: proline/hydroxyproline-rich glycoproteins
PAST: Pro, Ala, Ser, Thr
Pro/Hyp: proline or hydroxyproline
PRPs: proline-rich proteins
qRT-PCR: quantitative real-time PCR
RT-PCR: reverse transcription polymerase chain reaction
TAIR: The Arabidopsis Information Resource
WGT: whole genome triplication.
