# Supplementary material for: Genomic, Molecular Evolution, and Expression Analysis of Genes Encoding Putative Classical AGPs, Lysine-Rich AGPs, and AG Peptides in Brassica rapa
Source: Front Plant Sci. 2017 Mar 29;8:397. doi: 10.3389/fpls.2017.00397 (PMC5372829; doi:10.3389/fpls.2017.00397)
Supplement: Supplementary file 1 [file DataSheet1.doc]

# Genomic, molecular evolution, and expression analysis of genes encoding putative classical AGPs, lysine-rich AGPs, and AG peptides in *Brassica rapa*

# Running title: Putative AGPs in *Brassica rapa*

Tianyu Han, Heng Dong, Jie Cui, Ming Li, Sue Lin, Jiashu Cao, Li Huang

**Supplementary Methods**

Bioinformatics analysis was performed as the workflow diagram below:

**
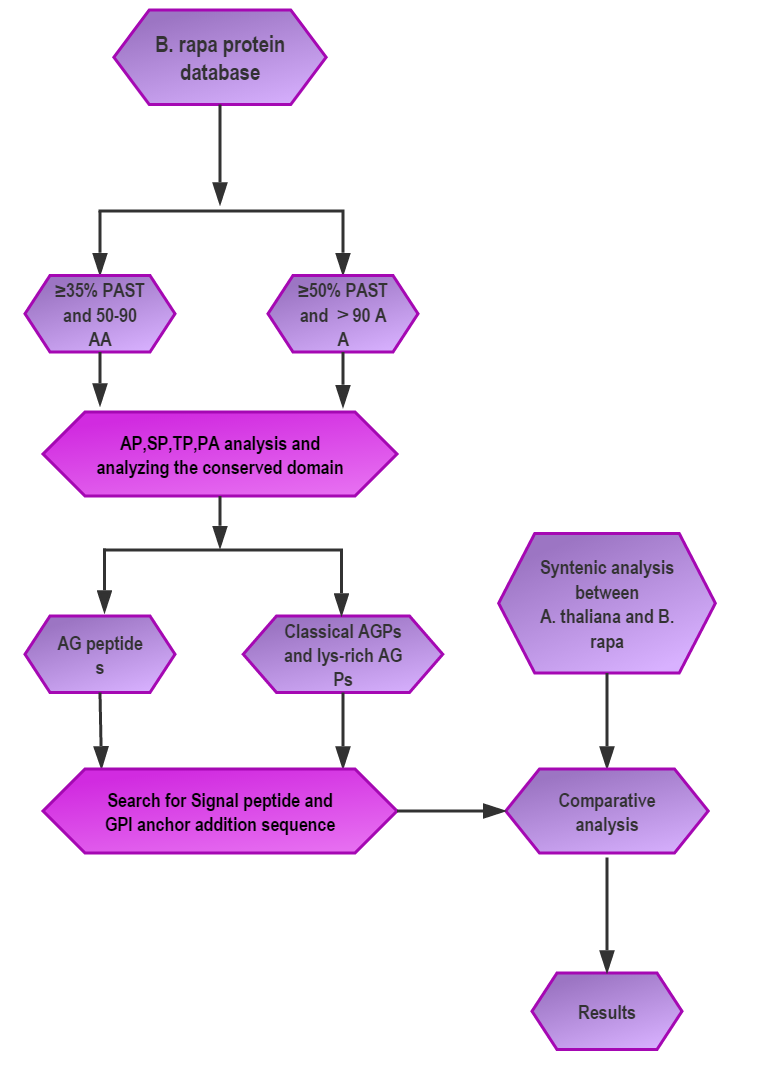
**

Perl Script was written as:

#!/usr/bin/perl

open (TXT,'C:\AGP\IRGSP-1.0_protein_2014-06-25.fasta'); # This part select your file that contain the whole protein sequences of your species.

open (IN,'>C:\AGP\Os35.fa');

#open (BI,'>C:\AGP\per.txt');

while (<TXT>) {

chomp;

if ($_=~/>(.+) \| Symbols.+/) {

$id=$1;

}else{

$hash{$id}=$hash{$id}.$_;

}

} # This part ranks each sequence in fasta format for the next calculation and output.

close TXT;

@ids=keys %hash;

foreach $id (@ids) {

$len=length($hash{$id});

if ($len>=50 && $len<=90) { # or $len>90 ,replace this line with if ($len>90) {

#print "$hash{$id}\n";

#print "OK\n";

$p=$hash{$id}=~tr/P/P/;

$a=$hash{$id}=~tr/A/A/;

$s=$hash{$id}=~tr/S/S/;

$t=$hash{$id}=~tr/T/T/;

#print "$p\n";

$num=$p+$a+$s+$t;

$per=$num/$len;

if ($per>0.35) { # or if ($per>0. 5)

#print "$id\n";

$exit{$id}=1;

}

}

} # This part selects the peptide under the proper length first. Then, calculation is taken to get the PAST percentage. After that, peptides is tested if they are fitting the requests.

open (TXT,'C:\AGP\IRGSP-1.0_protein_2014-06-25.fasta');

while (<TXT>) {

chomp;

if ($_=~/>(.+) \| Symbols.+/) {

#print "OK\n";

$id=$1;

if ($exit{$id}) {

#print "OK\n";

$i=1;

#print "$_\n";

print IN "$_\n";

}else{$i=0;}

}else{if ($i==1) {

print IN "$_\n";

}}

} # this part outputs the result operated above in order. The path can be changed in your requirement.

close TXT;

Accompanying description:

Detailed explain of each part was represented after the symbol #, and the description after symbol will not affect the script running. That means the description needs not to be deleted. The file suffix of perl script is pl, so save the codes as a file with the format of xxx.pl. The script hasn’t the program interface. In windows system, shell program cmd.exe can execute the script or double click the script program can execute the calculation. The output result is in the path you assigned.

**
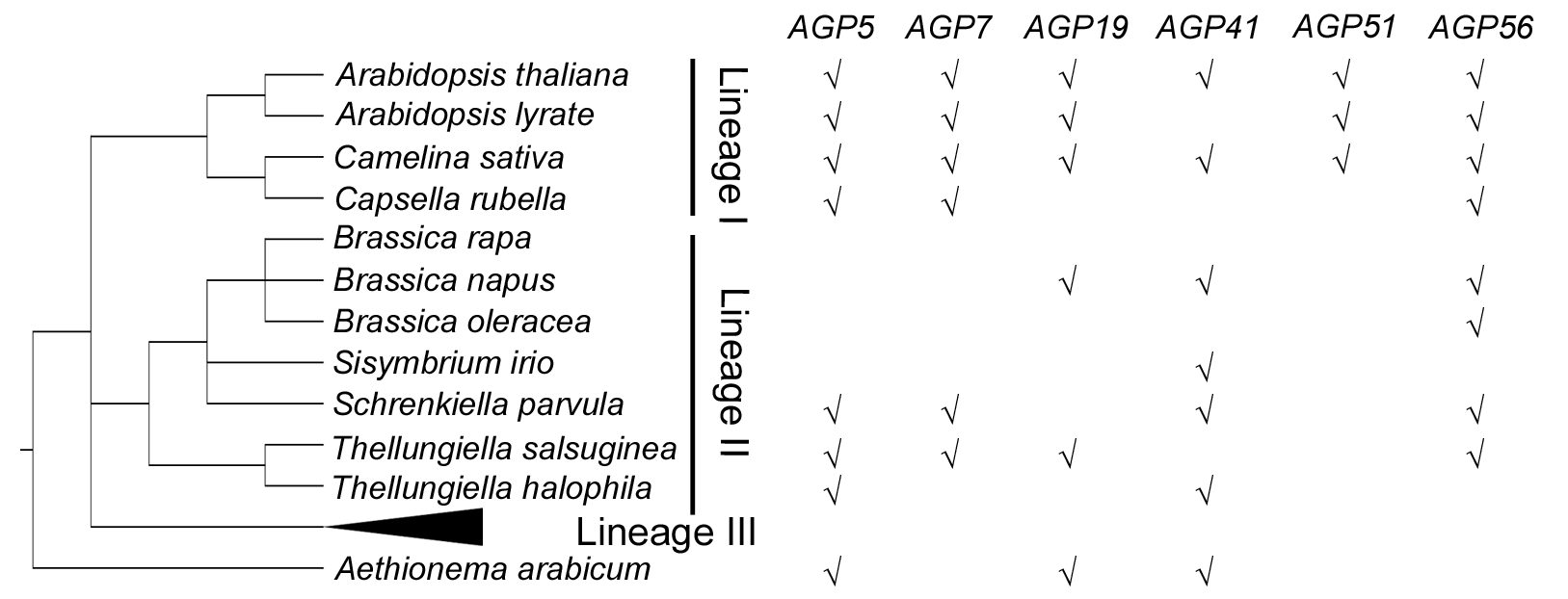
**

**Fig. S1 Orthologous genes of *AGP5*, *AGP7*, *AGP19*, *AGP41*, *AGP51* and *AGP56* in sequenced brassicaceae species.**

| **Table S1. Gene primers for RT-PCR analysis of *AGPs* in *Brassica rapa.*** | | | |
| --- | --- | --- | --- |
| Gene Name | Forward Primer Sequence (5'-3') | | Reverse Primer Sequence (5'-3') |
| *BrAGP1.1* | TTTGGCTATGTAGGTTCTTTG | | TATTTGCTTACCAGTAGGGAT |
| *BrAGP1.2* | TCTAATAAATCTCATTCCTCGAACC | | ATTCTTCTCGCTGCCATCTTC |
| *BrAGP2.1* | ACATCTCCTGTTGCATCTCCTCCTC | | AGCCAGCATCCATCTTCGTTCTTAC |
| *BrAGP2.2* | ACCAAAGGTCCGCTCAACTCA | | CAGCTCCAACTACTTCTCCAACAAC |
| *BrAGP3.1* | CTTCTCCTCCTGCTATGACACC | | TAAGCCAAAACGACAGCGTAC |
| *BrAGP3.2* | GCGACAGGGGAAGTTTTAGGA | | CAAGCTATGATCTTTCTTGGTCTAT |
| *BrAGP4.1* | CTCCTTCTCCCTCTGATGTTC | | AAAGCCCTTCCAATGGTAAAT |
| *BrAGP4.2* | GCGTCAACACTCGCTCAAGCT | | AGAGGCGGCGGGAACAACAGA |
| *BrAGP4.3* | AGAAATACTCACATAACACCAAAGA | | CTCCTCCTGTCGCTACTCCTC |
| *BrAGP6* | TCGTATTTGCTCTATTGGCT | | GTGGATGTGCTGTCGGGTGT |
| *BrAGP9.1* | AACTGTCTTGCTTGCTCCATT | | CGTATCGTATTTGCCGATTTT |
| *BrAGP9.2* | AGTTGGAACCATCATTTCATT | | CAAATTCGCTTTTAAGAGTAGA |
| *BrAGP10.2* | ATTGGACGCAACAACGCTATA | | TTGGGTTTGGTCAGAAAGATAAG |
| *BrAGP11.1* | CCACCACGCAGACCAAGGCA | | AATCCGACGGCGGCAACAGT |
| *BrAGP11.2* | TTTATCGTTTTTGCCCTTTT | | GTTGCCGAGTAATCATCTTC |
| *BrAGP12.1* | GCTTATGGTGGTGTTGATGG | | TTGGAGCTGGAGACTCTGTC |
| *BrAGP12.2* | CCCACCACAACTTCATCAACG | | GATGCCAAAGCAGCAACAGAT |
| *BrAGP13.1* | AAAAGCAGGGATAGACAAGGA | | TGGATGCAATGAAGATGAGAC |
| *BrAGP13.2* | TTAAACAATACATGAGTGAAAGCAG | | TGGAGGCAATGAAGATGAAAC |
| *BrAGP14* | CAGAGTCTCCAGCTCCAAGT | | AGAGTAATCCAACGACCAAA |
| *BrAGP15.1* | TACGAATCTCCATCGCAAAGA | | CTAACCACAAATGGCGTTCTC |
| *BrAGP15.2* | ATTTGTAAAACAAGCTCGTATAACG | | TACCGTCGCTTCAGCTCAGTC |
| *BrAGP15.3* | TCGAATCAAACTAAAACCCTA | | TCTGTACGAATCTCCATCACA |
| *BrAGP16.1* | TTTCGAGTGAAGATCAATCCC | | TCAACAATCAACAACGGCATA |
| *BrAGP16.2* | TTCGCCGTCGTCTCTTCTCT | | CACCAATGCCACCACCATTA |
| *BrAGP17* | TTCTACTCCAACCCCTTCAC | | TGTTCTTTCTACACCACTCT |
| *BrAGP18.1* | CTCGGCTCCACTCTGCATTAA | | CACATCCGCCAACGAAAAGGT |
| *BrAGP18.2* | CTCCGCTCTGCATTAATAATT | | CGACGATCAGGTACATTAGCT |
| *BrAGP20* | CCTGCTCCTTCTCCCACAAG | | CACCAAAGCCACCACCATTA |
| *BrAGP21.1* | GCCAAAGCAACAACAGAAG | | AATGGAGGCAAAGAAGATG |
| *BrAGP21.2* | CCACAGCTATTATCATCGCAACA | | GGTACGAACATGGCAGCATCA |
| *BrAGP21.3* | TAAATTCGTGTTCTTCTTCAA | | AAGACCAGATGCCAAAGCAAC |
| *BrAGP22.1* | CTTCGTCATTATCTCCGTGA | | AGAACATATGCTACCCCCTG |
| *BrAGP22.2* | AAGGACGCCATTCCTTCTGTT | | TTCTTTCACCAATCGGCATTT |
| *BrAGP22.3* | ACCTTCGCCAGCTCCAGCAC | | CAACGCCAAAGCCACCATCA |
| *BrAGP23.3* | GACCTGCAAGAACTTCTAAACA | | CACCAACCAACGAGCCAAGAG |
| *BrAGP24* | ACTCAACAAACTGCAACAAAT | | CACCAAATAAGGTAAACAACA |
| *BrAGP25* | CCTTCTACTCCAACCATTTCT | | TAGTGCTATAACGCCACAGAG |
| *BrAGP26* | AAGTTCCATAAACAAGCATAG | | AAACGTCTCCTTCTCCGTCAG |
| *BrAGP27* | AAAACAAGTGCGTGCGTGAGT | | GCCGACGACAAATACAATCCC |
| *BrAGP40.1* | ACCAGGTACTTCCTCCGCTAC | | AAGGCATTCCATAACATCACTTTAC |
| *BrAGP40.2* | CAATGAAGAATATCAACAAC | | CTGCAAGAGAAGAGCCAAGA |
| *BrAGP43.1* | AAAACCTTCCAGGTCTCAACT | | TCGTCTTCCGTCTTATTCACT |
| *BrAGP43.2* | AGATGAAGAAGATTGCCTGTG | | CTTCCGTCTTATTCACTTTGC |
| *BrAGP44* | GCCATGTTGATTATGGTTGC | | TGTTGCGGAGATTAGTTGTG |
| *BrAGP54.1* | CCAGCACATTCTCCTCTTCC | | TTTCTTCCATCCGCTCATCC |
| *BrAGP59* | TAAAACACCGACACCAGAATC | | AAACGGCAGCGTATAGAATAA |
| *BrAGP60* | TCTCCTTTGCAGCCTCCTGTC | | TCGCTGGTGCCGAACTACTCA |
| *BrAGP61* | ACACCTCAAGCAGCAGCAATG | | TCCACCGACTCAAATGACCAG |
| *BrUBC10* | GGGTCCTACAGACAGTCCTTAC | | ATGGAACACCTTCGTCCTAAA |
| **Table S2.** **Gene primers for qRT-PCR analysis of *AGPs* in *Brassica rapa.*** | | | |
| Gene Names | | Forward Primer Sequence (5'-3') | Reverse Primer Sequence (5'-3') |
| *BrAGP1.1* | | CATCTAGCGGAGGAAGGC | GAGAATCTGCGGAGGGTG |
| *BrAGP1.2* | | CAGCCGACGAATCAGAAG | TTCACGGTGGCTCAATCT |
| *BrAGP2.1* | | TCCTCCTCAAACCGATGC | ACGACGGCGTATAATGCT |
| *BrAGP2.2* | | CCAAAGGTCCGCTCAACT | CCGCATGGGCTAATCAAG |
| *BrAGP3.2* | | AGCTGGAGTTGGCTCGGTAA | GGTAGAGTCTCCTTCTCCCATTA |
| *BrAGP4.1* | | CTCCTTCTCCCTCTGATGTT | CACTTGGTGCAGGTGCTT |
| *BrAGP9.2* | | TCTAAAGTCTAGGGGTCC | AATTCGCTTTTAAGAGTAG |
| *BrAGP10.1* | | TGCAGCCGCGTTATTGTC | TCCACCGTCGCTTCACCA |
| *BrAGP10.2* | | GTTATGACACGCTTTACCT | AAAGTCTTGGGTTTGGTC |
| *BrAGP12.1* | | GTGGTGTTGATGGTGGCTAT | GCCGGGACGAACATAGTG |
| *BrAGP12.2* | | AGTCTCCAGCTCCAAGTC | AACAGAGGTAAAGGCAAA |
| *BrAGP13.1* | | AAGGAAGCGTCAGAGGTAGG | TCTTTGAGGCTGTCTTGGTG |
| *BrAGP13.2* | | GGGGATAGACAAAGAAGCA | TCAATGGAGGCAATGAAG |
| *BrAGP15.2* | | ACGAATCGCCATCACAAA | CGTCGCTTCAGCTCAGTC |
| *BrAGP16.2* | | AGCTGGTTTCGCGTTATTC | TTAGCAGATACGCAATCCCT |
| *BrAGP18.1* | | CTCGGCTCCACTCTGCAT | CCGCCAACGAAAAGGTAA |
| *BrAGP18.2* | | AAACCAAACAAGACTTTA | GACGATCAGGTACATTAG |
| *BrAGP21.1* | | GCCAAAGCAACAACAGAA | TGGAGGCAAAGAAGATGA |
| *BrAGP22.1* | | CATTGGAGATTCTCGCCGTCTT | TTCCATCGCTGGTGGGTGC |
| *BrAGP22.2* | | ATCTCCAACGGAAACTTCA | CTTTCACCAATCGGCATT |
| *BrAGP22.3* | | ACCTTCGCCAGCTCCAGCAC | CAACGCCAAAGCCACCATCA |
| *BrAGP25* | | ACGGCTCCTCGTACCCTGAC | GGCGGATGAGACAATGAGAAAG |
| *BrAGP26* | | CAAGATGTCAAAGAGGGCTTAC | GTCCACCAAATCCAGATGC |
| *BrAGP27* | | AGCGAGAAACAAGACACG | TTCCCTTTGAAGACCAGAT |
| *BrAGP40.1* | | CAGGTACTTCCTCCGCTAC | AAGGCATTCCATAACATCA |
| *BrAGP40.2* | | CAGCCACAGCGGCTACCAT | GAGCCAACCACCGGAAAA |
| *BrAGP43.2* | | GCTCTTGGCTCTTTGGTT | TTCCGTCTTATTCACTTTGC |
| *BrAGP59* | | CGGAGGAAGGCGAGTTAT | TTCTGATGGCGAGTCTGATAT |
| *BrAGP60* | | CAGCGACCCAGTCACCAT | CGACGCCCGTAAGGAAAT |
| *BrAGP61* | | GGCAAATACATAACAGCG | ATGACCAGGTCCGTAAAA |
| *BrUBC10* | | GGGTCCTACAGACAGTCCTTAC | ATGGAACACCTTCGTCCTAAA |

| **Table S3. The rate of molecular evolution in *AGPs* of *Brassica rapa.*** | | | | | | | |  |  |  |  |  |  |
| --- | --- | --- | --- | --- | --- | --- | --- | --- | --- | --- | --- | --- | --- |
| Classical AGPs | Ka | Ks | Ka/Ks |  | AG peptides | Ka | Ks | Ka/Ks |  | Lys-rich AGPs | Ka | Ks | Ka/Ks |
| *BrAGP1.1* | 0.2 | 0.6741 | 0.2967 |  | *BrAGP12.1* | 0.0481 | 0.2624 | 0.1833 |  | *BrAGP17* | 0.2376 | 0.5233 | 0.4541 |
| *BrAGP1.2* | 0.2006 | 0.5953 | 0.3369 |  | *BrAGP12.2* | 0.0318 | 0.2286 | 0.1393 |  | *BrAGP18.1* | 0.1539 | 0.4017 | 0.3832 |
| *BrAGP2.1* | 0.0877 | 0.3264 | 0.2688 |  | *BrAGP13.1* | 0.0732 | 0.5272 | 0.1388 |  | *BrAGP18.2* | 0.1164 | 0.3879 | 0.3001 |
| *BrAGP2.2* | 0.1147 | 0.3097 | 0.3703 |  | *BrAGP13.2* | 0.0813 | 0.4527 | 0.1795 |  |  |  |  |  |
| *BrAGP3.1* | 0.1172 | 0.5288 | 0.2217 |  | *BrAGP14* | 0.142 | 0.291 | 0.4881 |  |  |  |  |  |
| *BrAGP3.2* | 0.1619 | 0.5316 | 0.3045 |  | *BrAGP15.1* | 0.1371 | 0.6644 | 0.2064 |  |  |  |  |  |
| *BrAGP4.1* | 0.0907 | 0.4514 | 0.2008 |  | *BrAGP15.2* | 0.109 | 0.5316 | 0.2051 |  |  |  |  |  |
| *BrAGP4.2* | 0.1189 | 0.4659 | 0.2553 |  | *BrAGP15.3* | 0.1279 | 0.428 | 0.2988 |  |  |  |  |  |
| *BrAGP4.3* | 0.0853 | 0.488 | 0.1749 |  | *BrAGP16.1* | 0.0701 | 0.272 | 0.2578 |  |  |  |  |  |
| *BrAGP6* | 0.148 | 0.5145 | 0.2877 |  | *BrAGP16.2* | 0.0597 | 0.4816 | 0.1239 |  |  |  |  |  |
| *BrAGP9.1* | 0.1117 | 0.6706 | 0.1666 |  | *BrAGP20* | 0.076 | 0.2923 | 0.2601 |  |  |  |  |  |
| *BrAGP9.2* | 0.1051 | 0.4885 | 0.2151 |  | *BrAGP21.1* | 0.0611 | 0.4752 | 0.1285 |  |  |  |  |  |
| *BrAGP10.1* | 0.2097 | 0.3865 | 0.5426 |  | *BrAGP21.2* | 0.0343 | 0.4329 | 0.0792 |  |  |  |  |  |
| *BrAGP10.2* | 0.1675 | 0.6217 | 0.2695 |  | *BrAGP21.3* | 0.0608 | 0.5771 | 0.1054 |  |  |  |  |  |
| *BrAGP11.1* | 0.1435 | 0.5548 | 0.2586 |  | *BrAGP22.1* | 0.0374 | 0.2674 | 0.1398 |  |  |  |  |  |
| *BrAGP11.2* | 0.0875 | 0.3694 | 0.2369 |  | *BrAGP22.2* | 0.0607 | 0.239 | 0.2541 |  |  |  |  |  |
| *BrAGP25* | 0.1205 | 0.3501 | 0.3443 |  | *BrAGP22.3* | 0.0374 | 0.134 | 0.2791 |  |  |  |  |  |
| *BrAGP26* | 0.0949 | 0.2706 | 0.3507 |  | *BrAGP23.1* | 0.0553 | 0.212 | 0.2609 |  |  |  |  |  |
| *BrAGP27* | 0.131 | 0.2731 | 0.4798 |  | *BrAGP23.2* | 0.0472 | 0.3297 | 0.143 |  |  |  |  |  |
| *BrAGP50.1* | 0.128 | 0.3572 | 0.3583 |  | *BrAGP23.3* | 0.0393 | 0.3252 | 0.1207 |  |  |  |  |  |
| *BrAGP50.2* | 0.1493 | 0.3346 | 0.4462 |  | *BrAGP24* | 0.1448 | 0.6019 | 0.2406 |  |  |  |  |  |
| *BrAGP50.3* | 0.1288 | 0.3166 | 0.4068 |  | *BrAGP40.1* | 0.1009 | 0.2332 | 0.4326 |  |  |  |  |  |
| *BrAGP52* | 1.1594 | 1.2532 | 0.9251 |  | *BrAGP40.2* | 0.0492 | 0.2015 | 0.244 |  |  |  |  |  |
| *BrAGP53* | 1.1495 | 1.5824 | 0.7264 |  | *BrAGP42* | 0.3722 | 0.8997 | 0.4137 |  |  |  |  |  |
| *BrAGP54.1* | 0.2375 | 0.7763 | 0.306 |  | *BrAGP44* | 0.2332 | 0.4341 | 0.5373 |  |  |  |  |  |
| *BrAGP54.2* | 0.1388 | 0.5412 | 0.2564 |  | *BrAGP43.1* | 0.0235 | 0.2044 | 0.1149 |  |  |  |  |  |
| *BrAGP55* | 0.1582 | 0.5557 | 0.2846 |  | *BrAGP43.2* | 0.0395 | 0.2318 | 0.1473 |  |  |  |  |  |
| *BrAGP57* | 0.1431 | 0.7993 | 0.1791 |  | *BrAGP45* | 0.0739 | 0.3541 | 0.2087 |  |  |  |  |  |
| *BrAGP58.1* | 0.2084 | 0.6296 | 0.331 |  |  |  |  |  |  |  |  |  |  |
| *BrAGP58.2* | 0.1991 | 0.5414 | 0.3677 |  |  |  |  |  |  |  |  |  |  |
